# CIRCOAST: a statistical hypothesis test for cellular colocalization with network structures

**DOI:** 10.1093/bioinformatics/bty638

**Published:** 2018-07-19

**Authors:** Bruce A Corliss, H Clifton Ray, James T Patrie, Jennifer Mansour, Sam Kesting, Janice H Park, Gustavo Rohde, Paul A Yates, Kevin A Janes, Shayn M Peirce

**Affiliations:** 1 Department of Biomedical Engineering, University of Virginia, Charlottesville, VA, USA; 2 Department of Public Health Sciences, University of Virginia, Charlottesville, VA, USA; 3 Department of Biology, University of Virginia, Charlottesville, VA, USA; 4 Department of Ophthalmology, University of Virginia, Charlottesville, VA, USA

## Abstract

**Motivation:**

Colocalization of structures in biomedical images can lead to insights into biological behaviors. One class of colocalization problems is examining an annular structure (disk-shaped such as a cell, vesicle or molecule) interacting with a network structure (vascular, neuronal, cytoskeletal, organellar). Examining colocalization events across conditions is often complicated by changes in density of both structure types, confounding traditional statistical approaches since colocalization cannot be normalized to the density of both structure types simultaneously. We have developed a technique to measure colocalization independent of structure density and applied it to characterizing intercellular colocation with blood vessel networks. This technique could be used to analyze colocalization of any annular structure with an arbitrarily shaped network structure.

**Results:**

We present the circular colocalization affinity with network structures test (CIRCOAST), a novel statistical hypothesis test to probe for enriched network colocalization in 2D z-projected multichannel images by using agent-based Monte Carlo modeling and image processing to generate the pseudo-null distribution of random cell placement unique to each image. This hypothesis test was validated by confirming that adipose-derived stem cells (ASCs) exhibit enriched colocalization with endothelial cells forming arborized networks in culture and then applied to show that locally delivered ASCs have enriched colocalization with murine retinal microvasculature in a model of diabetic retinopathy. We demonstrate that the CIRCOAST test provides superior power and type I error rates in characterizing intercellular colocalization compared to generic approaches that are confounded by changes in cell or vessel density.

**Availability and implementation:**

CIRCOAST source code available at: https://github.com/uva-peirce-cottler-lab/ARCAS.

**Supplementary information:**

Supplementary data are available at *Bioinformatics* online.

## 1 Introduction

Interactions between vascular endothelial cells, which are arranged in arborized networks throughout all tissues of the body, and other cell types are instrumental in the initiation and perpetuation of a wide range of diseases, including diabetes mellitus (Ruggiero *et al.*, 1997). Interacting cell types with vascular endothelial cells include immune cells (Gerhardt and Ley, 2015), perivascular cells (Motherwell *et al.*, 2017; Sheets *et al.*, 2016) and stem cells (Amos *et al.*, 2008). Modulating intercellular interactions associated with disease progression is seen as a therapeutic target for preventing or ameliorating the associated pathology (Gartner *et al.*, 2017).

A key imaging-based measure of cell-cell interactions is intercellular colocalization, the frequency that two cell populations reside immediately adjacent to each other. Changes in intercellular colocalization suggest changes in cell-cell interactions and cellular behaviors that influence the interaction, including altered migrational capabilities, cytokine sensing and other chemotactic behaviors. Most research in cellular colocalization has focused on intracellular interactions with point-based features, specifically whether two molecular probes codistribute (dispersed in a spatially related fashion) or associate with a particular organelle (Dunn *et al.*, 2011). The statistics are often limited to a pixel-by-pixel analysis of correlation using Pearson’s correlation coefficient or Mander’s overlap coefficient (Zinchuk and Zinchuk, 2008), or more advanced analysis techniques such as spatial point pattern analysis (Burguet and Andrey, 2014; Helmuth *et al.*, 2010) or protein–protein interaction models (Johnson *et al.*, 2015). By contrast, there is a lack of statistical techniques to study cell–cell interactions (Payés *et al.*, 2012) where point-based analysis is less pertinent.

Cell populations are known to change dramatically in disease (Kowluru and Chan, 2008), which can confound metrics of colocalization. Intercellular colocalization events depend on the prevalence of the two interacting cell populations, and generic statistics cannot ascertain changes in colocalization because the data cannot be normalized to both cell populations simultaneously. This is especially problematic when there are substantial changes in vascular or cellular density between study groups or high variance between biological replicates. Here, we present an image analysis tool that statistically assesses intercellular colocalization independent of cell and network density by testing against a pseudo-null distribution for random intercellular colocalization events unique to each image. By comparing the intercellular colocalization fraction (ICF), the fraction of cells colocalizing with network structures, between an experiment image compared to the distribution of ICF values derived from modeling random cell placement in the same image, changes in colocalization can be ascertained relative to random behavior. Using additional statistics to combine data across images from a single biological replicate and compare between study groups yields a process that can characterize changes in intercellular colocalization affinity (ICA), which we define as the *frequency of colocation events between two cell populations corrected for changes in cell density, cell size and network density across study groups*.

An example where large changes in cellular density are observed is diabetic retinopathy, a disease that is marked by progressive damage to the retina (Wong *et al.*, 2016). Decreases in the densities of both blood vessels (Kowluru and Chan, 2008) and pericytes (Ejaz *et al.*, 2008), a cell type that colocalizes with and stabilizes the microvasculature, have been observed in early diabetes and are thought to initiate the degradation of the retina (Beltramo and Porta, 2013). Toward cell-based therapies, previous work has shown that injecting adipose-derived stem cells (ASCs) can ameliorate microvessel loss when ASCs colocalize with blood vessels and adopt a pericyte-like morphology in the retina and other tissues (Amos *et al.*, 2008; Mendel *et al.*, 2013). However, it was difficult to conclude whether or not ASC colocalization with the retinal microvasculature occurred at a rate greater than random chance without a validated statistical method.

We developed and validated CIRCOAST as a tool to measure intercellular colocalization by testing for a known enriched colocalization between ASCs and the arborized networks that endothelial cells form in culture. Then, we applied CIRCOAST to determine that locally delivered ASCs significantly colocalize with the retinal microvasculature in a murine model of diabetic retinopathy. By providing a robust method for evaluating the statistical significance of cell–cell colocalization, CIRCOAST provides insight into putative mechanisms of and potential therapies for a wide range of pathologies. This method naturally extends to the colocalization analysis of any annular shaped structure (disk-shaped such as a cell, vesicle or molecule) with any arbitrary background network structure within tissues or cells.

## 2 Materials and methods

### 2.1 Codebase

CIRCOAST was written in MATLAB 2016 b using the image processing toolbox and can be run either as source code or as compiled code with the MATLAB runtime environment version 9.1. The source code and compiled executable are available as a part of the Automated Random Cell Association Simulator (ARCAS) code repository (https://github.com/uva-peirce-cottler-lab/ARCAS). The user interface is designed with MATLAB’s graphical user interface development environment (guide), which allows the user to analyze a dataset of two or more color images, one marking the vasculature, and the others marking one or more cell types to be examined individually for enriched vascular colocalization. In this study, a confocal microscope was used to acquire a z-stack at approximately Nyquist sampling. The 3D images were then flattened with a max projection in the z axis dimension to produce a 2D RGB image.

### 2.2 Monte Carlo model development (MCMRP)

**Fig. 1.**
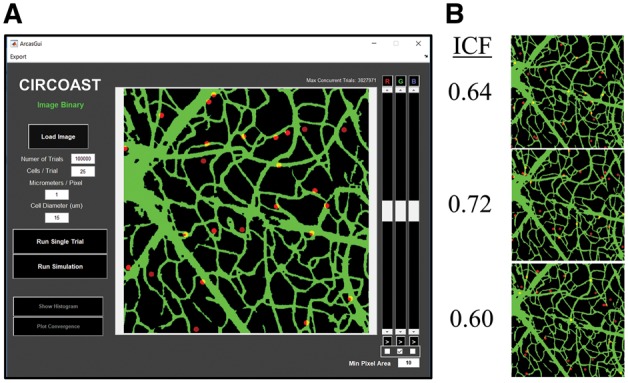
CIRCOAST GUI for analyzing cellular colocalization. (**A**) GUI for CIRCOAST that imports a thresholded vasculature and predicts the random cell colocalization fraction (ICF) (**B**), through a series of trials from a Monte Carlo model of random placement (MCMRP)

An initial Monte Carlo model of random placement (MCMRP) was created to simulate randomly placed cells. An input image of the network is imported into the CIRCOAST GUI, and segmented via an adjustable global threshold (Fig. 1A). Image resolution, cell size and number of cells are set by the user, and a series of Monte Carlo simulations are performed with an agent-based model that stochastically spawns cells in an image, in which under the random cell placement paradigm, every location within the input image region has an equal chance of being selected as a site of cell placement; and once placed, the fraction of cells overlapping with the vasculature network is calculated (Fig. 1A, ICF). A probability distribution for the ICF in a given image is approximated based on the thousands of Monte Carlo simulation repetitions of the random cell placement process (detailed outline of algorithm in Supplementary Note 1).

Probing of the key parameters that influence cellular colocalization with the vasculature was undertaken via a simple program created to stochastically create a network structure resembling blood vessels in a controlled manner (Supplementary Fig. S1). Dense microvascular networks have been described as having structural characteristics of interconnected wires (Longden *et al.*, 2017). The stochastically-generated networks were created by defining a randomly seeded point cloud with an enforced minimum distance between points, and using the watershed algorithm to create a network of line segments bisecting all points. Line segments were then iteratively removed until the desired vessel network was obtained, mimicking the range of vessel density found in vascularized tissues (extended explanation of algorithm in Supplementary Note 2).

A set of parameters defining the network structure and the cell-of-interest (COI) were identified (Supplementary Fig. S2), including: (i) network fraction: the fraction of pixels in an image that comprise the network, (ii) network length density: the length of the centerline for all network structures divided by the area of the image, (iii) network radius: thickness of network orthogonal to the network centerline, (iv) cell number, (v) cell diameter and (vi) cell-dilated network fraction (CDNF). CDNF defines the area of the image where if the center of a COI is within that area, the COI overlaps with the network by at least one pixel and is counted as being a colocalized cell, which is captured by morphologically dilating the segmented network with the length of the radius of the COI. Therefore, with a fixed network structure, as cell diameter increases so will the value for CDNF.

The relation between each system parameter and the ICF predicted by the MCMRP was examined over a wide range of parameter values (Supplementary Fig. S3). All parameters correlated with the MCMRP derived mean ICF except for cell number, suggesting many variables influence the mean of the ICF distribution under the random cell placement paradigm, but giving little insight to what parameter(s) directly dictate network colocalization.

**Fig. 2.**
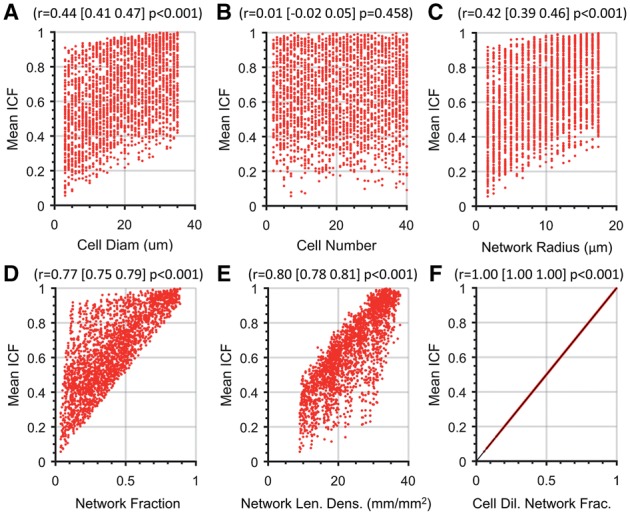
Network area fraction dilated by cell radius determines the random cell colocation fraction. The mean ICF was calculated with the MCMRP over 10 000 trials with randomly selected parameters and displayed as a function of (**A**) cell diameter, (**B**) cell number, (**C**) network radius, (**D**) network fraction, (**E**) network length density and (**F**) cell-dilated network fraction (CDNF, *N*=2 500 images). Pearson correlation coefficient and associated 95% confidence interval and *P*-values are provided at the top of each scatterplot

To determine if any of the parameters can directly predict mean ICF under the random cell placement paradigm, a dataset of 2500 simulated experiments were generated using the vessel network generator with randomly assigned parameters. The means of ICF distributions derived from the 2500 experiments were correlated with the individual parameters (Fig. 2). While most parameters correlated with mean ICF, only CDNF had a correlation coefficient (*r*) of 1 (rounded to within 6 decimal places), suggesting that CDNF correlates almost perfectly with the MCMRP derived mean ICF (Fig. 2F). Note that in Figure 2F; that for all intended purposes, the relationship between the CDNF values and mean ICF values is deterministic (points fall on a 45° line).

To further examine if CDNF is a unique predictor of ICF, a multivariable linear regression (MVLR) analysis was conducted with all input parameters as predictor variables and mean ICF as the response variable. Input parameter values and mean ICF values were converted to *z*-scores so that all of the multivariate regression model coefficients shared the same scale of measure while still preserving the underlying multivariate relationships that exist on the non-*z*-score scale. All predictors had insignificant *P*-values except for CDNF (Table 1). Furthermore, since the regression coefficients of all predictors other than CDNF were essentially equal to zero, CDNF is the only input parameter that was given any weight in the MVLR in terms of predicting the mean ICF *z*-score. Given that the MVLR model multiple coefficient of determination (*R*^2^) was =1, we conclude that CDNF is highly and uniquely correlated with the predicted mean ICF of the MCMRP. Although the ICF in given MCMRP trial can differ from CDNF due stochasticity, and the ICF from an acquired image may differ from CDNF from stochasticity or non-random cell placement, the CDNF can be used to calculate the mean ICF *from random behavior* in both cases.

**Table 1.** Multivariable regression of z-scored input parameters versus the z-score of the ICF predicted by Monte Carlo model of random placement (*R*^2^=1.00)

Predictor	Coefficient	SE	F-statistic	P-value
Intercept	−9.54E-16	1.20E-05	—	—
Cell diameter	−1.96E-05	2.56E-05	−0.76	0.444
Cell number	−5.57E-06	1.20E-05	−0.46	0.642
Network Rad.	−2.59E-05	4.34E-05	−0.60	0.551
Network Frac.	5.01E-06	5.74E-05	0.09	0.931
Network. Len. Dens.	−3.24E-05	4.67E-05	−0.69	0.489
**C**DNF	**1.000**	**5.00E-05**	**2.00E4**	**<0.000**
Model	—	—	1.16E9	<0.000

*Note*: *z*-score transformation preserves the underlying multivariate relationships. Significant predictors highlighted in bold.

### 2.3 Binomial model development and validation (BMRP)

Based on the aforementioned Monte Carlo findings, CDNF was used to develop a binomial model of random placement (BMRP) for a more mechanistic and exact representation of cell colocalization under the random placement paradigm. By defining CDNF as the probability of success for a randomly placed cell colocalizing with the vasculature within an image, intercellular colocation can be modeled as a binomial stochastic process using Equation (1) (Sokal and Rohlf, 2012);

(1) fc;n,p=ncpc1-pn–c,

where *p* is the cell-dilated network fraction (CDNF), *c* is the number of cells colocalizing and *n* is the total number of cells in the image. The mean (*μ*) and standard deviation (*σ*) of the distribution can be directly calculated using the formulas listed in Equation (2) (Sokal and Rohlf, 2012), as opposed to approximating the values based on successive MCMRP trials.

(2) $$\mu =n\times p,\;\sigma =\sqrt{n\times p\times \left(1-p\right)}\;$$

**Fig. 3.**
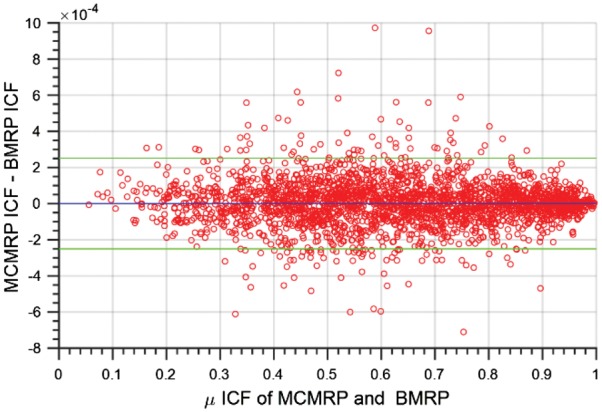
Discrepancy between BMRP predicted mean ICF and MCMRP predicted mean ICF. Bland Altman plot of the 2500 pairs of BMRP and MCMRP predicted mean ICF values, with the difference in paired values plotted against the average. Note that the blue horizontal line identifies the mean discrepancy between the 2500 pairs of BMRP and MCMRP predicted mean ICF values (mean: 1.05E-7), and the green horizontal lines identify the lower and upper 95% confidence limits (−0.00025, 0.00026) for the discrepancy between any pair of BMRP and MCMRP mean ICF values

To evaluate the BMRP, the predicted mean ICF from random cell placement was compared to the MCMRP predicted mean ICF (10 000 trials/image) across the same dataset of 2500 simulated experiments that were used to produce Figure 2. The discrepancies between the predicted mean ICFs of the BMRP and the MCMRP are shown as a function of the predicted mean ICF in Figure 3.

**Fig. 4.**
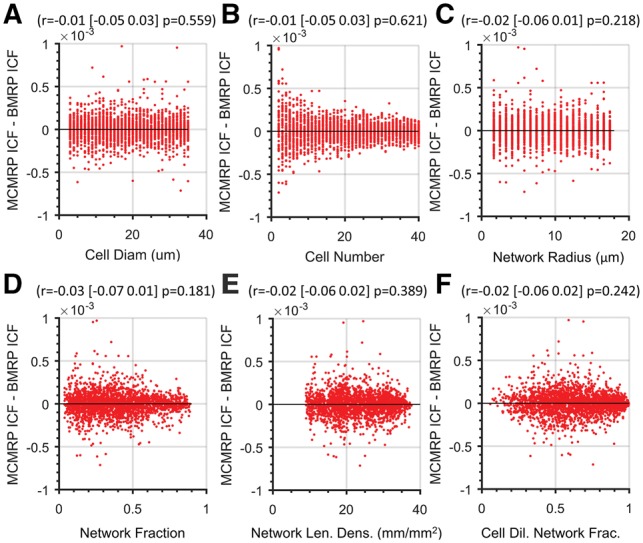
Discrepancy between BMRP mean ICF and MCMRP mean ICF versus the cell and network input parameter values. (**A–F**) Relationship between each input parameter and the discrepancy between the BMRP predicted mean ICF values and the MCMRP predicted mean ICF values. Pearson correlation coefficient and associated 95% confidence interval and *P*-value are provided at the top of each scatterplot

A paired two-tailed *Student’s t*-test revealed no difference between the predicted mean ICFs of the BMRP and the MCMRP (*P* = 0.850, *α* = 0.05). Furthermore, no systematic relationship could be detected between the discrepancy between the predicted mean ICFs of the BMRP and the MCMRP as a function of the mean of predicted model ICFs (*P* = 0.248, Pearson correlation), nor were any of the input parameters individually systematically related to the discrepancy between the predicted mean ICFs of the BMPR and the MCMRP (Fig. 4A–F). When the *z*-scores of the input parameters were used as predictor variables in a MVLR model to predict the *z*-score scaled values for the discrepancies between the predicted mean ICFs of BMPR and MCMRP, neither the MVLR model nor any of the input parameters individually were significant predictor(s) of the ICF discrepancy *z*-score (Table 2).

**Table 2.** Multivariable linear regression of *z*-scored input parameters versus the *z*-score of the difference in mean ICF predicted by MCMRP and BMRP (*R*^2^<0.00)

Predictor	Coefficient	SE	F-statistic	P-value
Intercept	−0.013	0.017	—	—
Cell diameter	−0.023	0.036	0.40	0.526
Cell number	0.017	0.017	1.05	0.305
Network Rad.	−0.100	0.060	2.74	0.098
Network Frac.	0.100	0.799	1.55	0.213
Net. Len. Dens.	0.048	0.065	0.54	0.462
CDNF	−0.004	0.070	0.00	0.968
Model	—	—	1.38	0.217

With no significant difference seen between the mean ICF values predicted by the BMRP and the MCMRP, and no systematic relationships seen in the discrepancies between the predicted mean ICFs of the BMPR and the MCMPR across the input parameter space, we concluded that the BMRP accurately represents the MCMRP, and that random cell placement can be modeled as a binomial stochastic process using Equations (1) and (2).

### 2.4 Hypergeometric model and validation (HMRP)

The binomial model assumes that the random placement of cells is completely independent events, that each successive COI placed in an image can be placed anywhere. However, in practice, COIs cannot overlap because they would be counted as a single cell. Thus, at higher cell densities, there are less locations for additional cells to be added and still counted as additional cells in the image, suggesting that placement of cells are related events and there exists a maximum number of placed cells, possibly suggesting that a hypergeometric model of random placement (HMRP) could be more suitable to model this process:

(3) hk|N,n,K=KkN-Kn-kNn

where *k* is the number of colocalizing cells, *n* is the total observed number of cells in the image, *N* is the max number of cells that can exist in the image, *K* is number of colocalizing cells of the max population of cells placed in the image. The mean (*μ*) and standard deviation (*σ*) of the distribution can be directly calculated using the formulas listed in Equation (4) (Sokal and Rohlf, 2012):

(4) $$u=\frac{n\times k}{N}\;\sigma =\sqrt{\frac{n\times k\times \left(N-k\right)\times \left(N-n\right)}{N^{2}\times \left(N-1\right)}}$$

Theoretically, the max number of non-overlapping cells that can be found in an image is defined by the hexagonal packing of circles, which previous research has shown to have a packing ratio of 0.901 (fraction of image area covered by circles), demonstrated to be invariant to the size of the circle and bounding area (Steinhaus, 1999). Yet this packing assumes perfect placement of all circles. Randomly placed non-overlapping pixelated cells may have a much lower packing ratio than this scheme, and may not be invariant to cell size since the shape of a cell is approximated in a pixelated fashion. Moreover, cells must be fully contained in the image area to contribute to the packing ratio for possibly colocalizing cells; cells that exceed the border of the image are not counted because their colocalization state with the network outside of the image cannot be determined.

We designed a Monte Carlo model of random cell placement without replacement that iteratively places cells until no more can fit in the image and then calculates the final packing ratio. Doing this for a range of cell pixel diameters reveals that the packing ratio of randomly placed fully contained non-overlapping pixelated circles changes with cell size (*P* = 0, Kruskal Wallis, *N* = 100 trials, Supplementary Fig. S4A) and image dimensions (*P* = 0, Kruskal Wallis, *N* = 100 trials, Supplementary Fig. S4B). For cell diameters <5 pixels, distinct cells cannot be discerned at the highest density, so experiment data with that low cellular resolution is considered invalid. For cell diameters above five pixels in diameter, a look up table is provided (Supplementary Table S1) for 512 by 512 image dimensions and is used for calculating the *N* parameter in a hypergeometric distribution:

(5) $$N=\frac{A*\eta }{\pi r^{2}}$$

where *A* is the pixel area of image, *η* is the packing ratio from the look up table and *r* is the pixel radius of the cell [Equation (5)]. The CDNF of each image is used to approximate the number of cells colocalizing (*k*) from the max population of cells (*N*), since it represents the fraction of the image where colocalization occurs:

(6) k=Np

where *p* is the CDNF, used also in the binomial distribution from Equation (1).

The mean ICF from the BMRP was compared to HMRP with the same dataset in Figure 3. No difference was seen in mean ICF values (*p* = 0.194, paired *t*-test, *N* = 2500, Supplementary Fig. S5A). Multiple variable linear regression revealed a marginally significant relationship between cell diameter and CDNF (Supplementary Fig. S5B–H), but the magnitude of the discrepancy between the models was negligible (mean difference: 4.26E-6), on par with difference between the MCMRP and BMRP models. The BMRP was selected for experimental use since its discrepancy with the HMRP mean ICF was negligible. Furthermore, with the HMRP, the parameter *N* changes with both cell size and image size: the computational demand of running simulations to approximate the max cell number in a given image makes it impractical to present as a general method until these parameters can be calculated in a more efficient and parameter invariant fashion.

Related to the issue that placement of cells are dependent events is whether homotypic interactions of the COI (cells migrating based on the position of other cells of the same type to form clumps) would alter the ICF. Encouragingly, we found that there is no difference in mean ICF from random placement of individually placed cells compared to cells placed in non-overlapping or overlapping clumps, suggesting that colocalization with the network structure is independent of self-colocalization with the COI (Supplementary Fig. S6).

### 2.5 Statistical pipeline

Statistical processes were created to test for: (i) enriched ICA of a cell type with the network structure within a single image, (ii) enriched ICA for a study group of images and (iii) unique ICA between two study groups. All three of these tests were conducted by examining where the observed value of the random variable is located along the null probability distribution (Supplementary Fig. S7).

#### 2.5.1 CIRCOAST test: testing colocalization for single image

To test for enriched colocalization affinity in a given image, the network structure in the image is thresholded and segmented, dilated by the radius of the COI, and the fraction of white pixels defines the cell-dilated network fraction for that image. Under the binomial stochastic model, the CDNF and cell number is used to calculate the probability of observing colocalization with the network to an equal or greater extent than what is observed in the image if colocalization occurs under random placement (Supplementary Fig. S7A). Equation (3) is utilized to derive the *P*-values for a one-tailed binomial hypothesis test:

(3) CIRCOAST p=1-∑cnncpc1-pn-c

where *c* is the observed number of cells colocalizing in the image, *n* is the total number of cells in the image and *p* the cell-dilated network fraction (CDNF) for that image. The null hypothesis that the image exhibits a degree of colocalization no greater than what would be expected by chance is rejected if CIRCOAST *p* ≤ 0.05.

Since intercellular colocalization is modeled as a binomial process, sufficient sampling is determined by the quantity of each cell population sampled rather than fields of view imaged. In order to ensure that sufficient sampling can be obtained for each biological replicate, the data from multiple images is pooled together by calculating the combined CDNF across images and summing total COIs found in each image. This technique was validated by comparing the CDNF and CIRCOAST p-value calculated from a test image compared to splitting it into four sub images. No difference was seen between the test images and original image, confirming the validity of this technique to join colocalization information across images (Supplementary Fig. S8). Notably, while this process can probe for enriched colocalization affinity in an image, it fails to directly test for enriched colocalization for a study group of multiple biological replicates (animals, culture well plates, etc.).

#### 2.5.2 One-sample CIRCOAST test: testing colocalization for a single study group

We assert that a statistical test that probes for enriched colocalization within one or more study groups requires a random variable that acts as a metric of colocalization and is scaled to reflect the degree of colocalization beyond what would be expected purely by chance. The *P*-value from the CIRCOAST calculated from the binomial hypothesis test in Equation (3) satisfied both of the aforementioned criteria. *P*-values are known to be reliable random variables suitable for hypothesis testing (Sackrowitz and Samuel-Cahn, 1999). The mean CIRCOAST *P*-value is calculated across the joined images of each animal or subject (Supplementary Fig. S7B). The null distribution of the mean CIRCOAST *P*-value is simulated by assuming the null hypothesis is true and approximating what the distribution of the mean CIRCOAST *P*-value would be under the random placement paradigm (i.e. the *pseudo*-null distribution). When the null hypothesis is true, the *P*-value of a hypothesis test is a uniform (0, 1) random variable. Therefore, to generate a pseudo-null distribution for the mean binomial *P*-value, sets of *N P*-values are generated from a uniform (0, 1) distribution to match the *N* number of biological replicates in the experiment data and the mean of the distribution of generated *P*-values is calculated. Repeating this process (10 000 000 trials) yielded the *pseudo-*null distribution of the mean CIRCOAST *P*-value under the random cell placement paradigm. The percentile at which the observed mean *P*-value falls along the distribution of simulated mean *P*-values yielded a *one-sample cellular colocalization affinity with network structures P*-value (1-sample CIRCOAST) for enriched colocalization across the entire study group by combining the information from the unique binomial distributions found in each image.

#### 2.5.3 Two-sample CIRCOAST test: testing colocalization between two study groups

To determine if two study groups differ with respect to the frequency of cell–cell colocalization, the CIRCOAST *P*-values are calculated for all the images from each group and subjected to a two-sample parametric (e.g. Student’s *t*-test) or non-parametric (e.g. Wilcoxon rank sum test) test to yield an ‘observed’ *P*-value (Supplementary Fig. S7C). This *P*-value is then compared to the pseudo-null distribution of *P*-values that are generated under the null hypothesis scenario. The pseudo-null distribution is generated by way of a large number of permutations of the study group identifications; i.e. the original study group identifications are randomly assigned to the sample identification numbers and the same parametric or non-parametric two-sample test is conducted using these random study group assignments. The fraction of the two-sample permuted test *P*-values less than or equal to the ‘observed’ two-sample test *P*-value yields the *two-sample cellular colocalization affinity with network structures P*-value (2-sample CIRCOAST).

### 2.6 Experimental validation

#### 2.6.1 *In silico* validation

A dataset of images was created to represent healthy and diseased tissue, with one study group with high injected cell and endothelial cell network density to mimic healthy conditions, and the another with low injected cell and endothelial cell network density for the dropout seen in diabetes (Supplementary Fig. S9). Vessels were created with the vessel network generator program and cells randomly placed with a single run of the MCMRP. Correct statistical analysis should reveal no changes between study groups since the cells were randomly placed. While generic statistics revealed a change in ICA (colocalization per field of view: *p* = 2.46e-09; colocalization per 1 mm vessel length: *p* = 1.12e-02; fraction of injected cells colocalizing: *p* = 6.68e-07; unpaired *t*-test), CIRCOAST correctly revealed no changes in colocalization between study groups (*p* = 0.494). This dataset reveals that a false positive conclusion can be generated by generic statistics tests that confound changes in vascular and cell density when examining intracellular colocalization.

Imaged cells have a range of phenotypes in both size and shape that depart from the idealized uniform disk shape used in the MCMRP. We determined that a collection of cells with heterogeneous diameters can be approximated as cells with diameter equal to the mean diameter, yielding mean ICF values that are not identical, but have close agreement and negligible effect sizes (Supplementary Fig. S10). While the shape used to represent a cell can alter the ICF from random cell placement, representing cells as disk whose area is equal to the mean cell area sampled from a collection of imaged cells minimizes inaccuracies from altered geometry (Supplementary Fig. S11).

Errors in analyzing experimental images, such as failing to identify all cells in an image or discerning individual cells from cell clumps, could potentially throw off results of the CIRCOAST test. To examine the consequence of input error, we created a dataset of 2000 simulated images, split into 50 study groups with 20 images each, to see how errors in quantification alter mean ICF, CIRCOAST 1-sample P, and statistical outcome. Simulated images had uniform vascular density and elevated cell density to induce a high degree of cell overlap with randomly placed cells.

With highly erroneous quantification represented by cell count quantified by connected components (any overlap between cells leads to them being counted as a single cell, roughly 20% of cells miscounted), CIRCOAST 1-sample *P*-values were significantly reduced, leading to an elevated false positive rate (Supplementary Fig. S12A–E). However, this effect was mitigated by calculating the input cell area using the diameter approximated circle method. Therefore, the error caused by incorrectly quantified cell clumps can be minimized by accounting for how the cell clusters change the mean cell area, although high emphasis should be placed on correct cell counting in experiment images. If cells are randomly missed and not counted in the quantification process, mean ICF or CIRCOAST 1-samples *P*-values do not change (Supplementary Fig. S12F–H), corroborated by the fact that cell density does not change mean ICF with the MCMRP parameter sweeps in Supplementary Figure S3.

#### 2.6.2 Cell sources

See Supplementary Note 3.

#### 2.6.3 *In vitro* and *in vivo* validation

See Supplementary Note 4.

#### 2.6.4 Image acquisition, thresholding and quantification

See Supplementary Note 5.

## 3 Results

A biologically relevant application of colocalization of annular cells with network structures is cellular colocalization with microvascular networks, which are comprised of branched networks of endothelial cells. ASCs are known to colocalize with vascular endothelial cells *in vitro* (Merfeld-Clauss *et al.*, 2010) and can engraft when injected *in vivo* (Mendel *et al.*, 2013). Active homing of ASCs to the vasculature is hypothesized to play a role in this intercellular colocalization, but has not been established at a cell population level. The CIRCOAST statistical pipeline is validated by testing for the enriched cellular colocalization known for ASCs and ECs *in vitro* compared to fluorescent microspheres for a negative control, and then used *in vivo* to determine if injected ASCs exhibit greater than random colocalization with the vasculature to give insight to their possible mode of therapeutic action in disease (Mendel *et al.*, 2013).

**Fig. 5.**
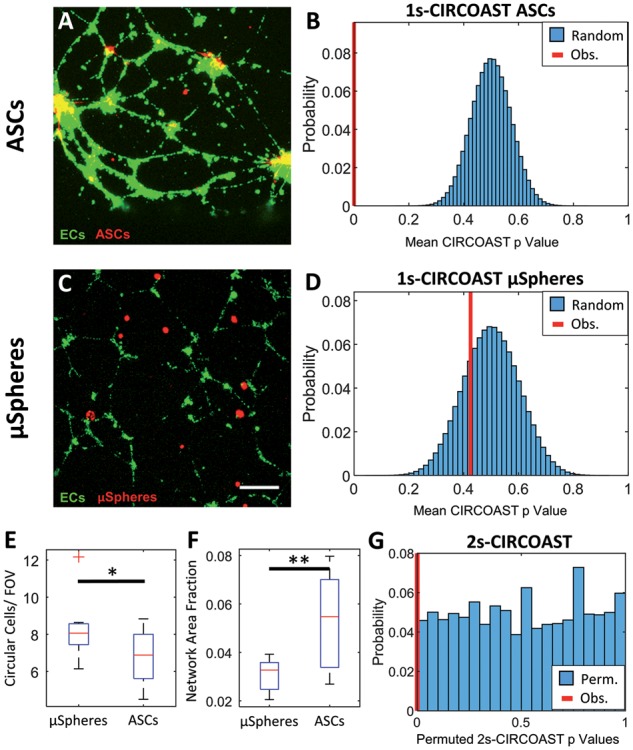
ASCs exhibit enriched colocalization with HUVECS network, while fluorescent microspheres (μSpheres) do not. (**A**) ASCs (red) co-cultured with HUVECS (green). (**B**) Distribution of simulated mean CIRCOAST *P*-values (blue) of random colocalization of ASC group compared to observed mean CIRCOAST *P*-value (red). (**C**) Fluorescent μSpheres seeded on a culture of HUVECs (scale bar 250 um). (**D**) Distribution of simulated mean CIRCOAST *P*-values (blue) of random colocalization from fluorescent μSpheres compared to actual mean *P*-value (red). (**E**) Circular cell density and (**F**) endothelial network density between groups. (**G**) Distribution of *P*-values (blue) derived from permuting CIRCOAST *P*-values in a Wilcox sum rank test between ASCs and μSpheres, with observed *P*-value (red) (*N*=6 wells, 3 images/well)

ASCs cultured with a network of HUVECS were found to have enriched colocalization over random behavior predicted by the BMRP (Fig. 5A and B;*p* < 1e-7 1-sample CIRCOAST, *α*= 0.05, 1e7 trials, *N* = 6 wells, 3 images/well). For a negative control, fluorescent microspheres cultured with a network of endothelial cells did not exhibit unique colocalization as expected (Fig. 5C and D;*p* = 0.235 1-sample CIRCOAST, *α* = 0.05, 1e7 trials, *N* = 6 wells, 3 images/well). There was a significant difference in ASC and microsphere cell density (Fig. 5E, −18.7%, *p* = 2.4E-2 two-sample *t*-test) and endothelial network density (Fig. 5F, +74.5%, *p* = 3.3E-3 two-sample *t*-test) between study groups, illustrating the need for statistical tests that are not confounded by changes to cell or vessel density across study groups. As demonstrated in Supplementary Figure S9, changes in network and cell density can confound generic statistics that examine colocalization events, while the CIRCOAST takes into account these changes between study groups and effectively standardizes for both changes in network and cell density. Unique colocalization affinity between ASCs and microspheres with endothelial cells was detected (Fig. 5G, *p* = 1.3e-6, 2-sample CIRCOAST, *α* = 0.05, 1e7 permutations).

**Fig. 6.**
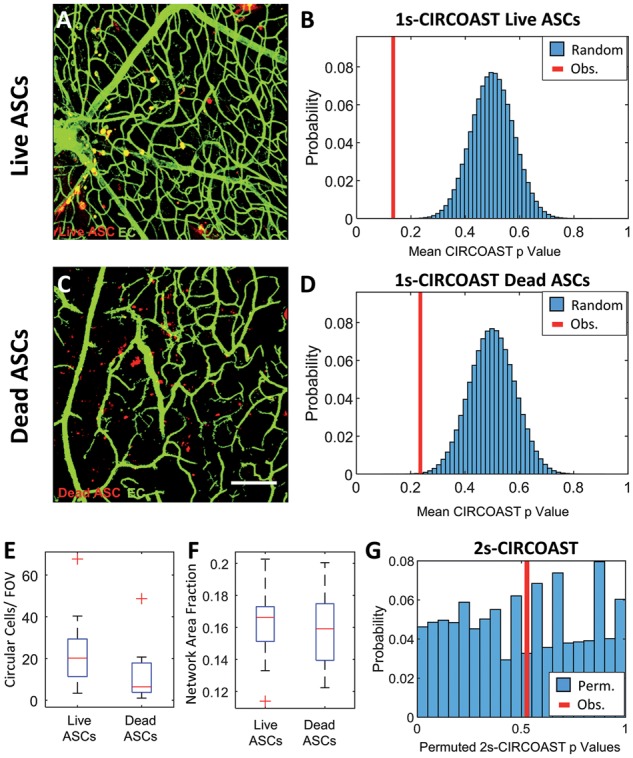
Injected live and dead ASCs both exhibit enriched intercellular colocalization affinity with the vasculature. (**A**) Confocal image of retinal vasculature (green, preprocessed and thresholded) and injected with live DiI-labeled circular ASCs (red). (**B**) Distribution of simulated mean CIRCOAST *P*-values (blue) of random colocalization of ASC group compared to observed mean binomial *P*-value (red). (**C**) Dead DiI-labeled circular ASCs in the retinal vasculature (scale bars 150 um). (**D**) Distribution of simulated mean CIRCOAST *P*-values (blue) of random colocalization from dead cell group, compared to actual mean *P*-value (red). (**E**) Injected circular cell and (**F**) endothelial network density between study groups. (**G**) Distribution of permuted *P*-vales of Wilcox sum rank test of CIRCOAST *P*-values between study groups, with observed *P*-value (red) (*N*=6 mice, 3 images/mouse)

For an *in vivo* validation of the CIRCOAST test, ASCs were injected into the eye in an *in vivo* model of diabetic retinopathy and found to exhibit enriched colocalization with the retinal vasculature (Fig. 6A–B, *p* < 2.0e-7 1-sample CIRCOAST, 1e7 trials, *N*= 6 wells). Surprisingly, injected dead cells also had enriched colocalization with the vasculature (Fig. 6C–D, *p* < 5.2e-4 1-sample CIRCOAST, 1e7 trials, *N* = 6 mice, 3 images/mouse), possibly due to immune cells phagocytosing the injected dead cells while still retaining their fluorescent signal (Kang *et al.*, 2014; Sutton *et al.*, 2008), and then chemotaxing to the vasculature and reentering the bloodstream (Cortez-Retamozo *et al.*, 2012). While there was no change in EC density (Fig. 6F, 2.24%, *p* = 0.70 two-sample *t*-test), there was a trend of decreased injected circular cell density in the dead cell group (Fig. 6E, −48.2%, *p* = 0.071 two-sample *t*-test), and a high degree of variance between biological samples, illustrating the need for statistical tests to correct for high variance with cell densities. No difference was discerned in colocalization affinity between live and dead ASCs (Fig. 6G, *p* = 0.53 2-sample CIRCOAST, 1e7 permutations).

## 4 Discussion

In summary, we present CIRCOAST, a tool to characterize intercellular colocalization with network structures independent of the changes in cell and vessel network density found across study groups from both *in vitro* and *in vivo* experiments. The tool was validated by probing for the previously known colocalization events observed between ASCs and endothelial cells *in vitro*, and used to test for enriched colocalization between these cells *in vivo*.

In the field of immunology, changes in cell density measured via flow cytometry or fluorescence microscopy are used as key metrics to study cell behaviors in disease (Krummel *et al.*, 2016). These measurements report cell numbers or densities and only indirectly allude to changes in cell–cell interactions. In studies when cellular colocalization is examined more directly using microscopy, changes to cell density of either cell population can confound colocalization metrics analyzed with generic statistics: the method presented here does not have such drawbacks. Additionally, this method could be used to test for changes in colocalization within subpopulations of a single cell type denoted by unique marker expression to implicate marker expression with colocalization behavior.

Although we focused on characterizing the frequency of interactions between cells and microvessels, other static cellular network structures could be analyzed, such as neuron networks, glial cells and lymphatics. Furthermore, we think that CIRCOAST could be extended beyond cell-vessel associations to study colocalization between two migrating cell populations so as to interrogate putative chemotactic behaviors. Possible applications include the study of interactions between T-cells and B-cells, which are known to be critical for T-cell activation and immune responses to infection (Janeway *et al.*, 2001). Additionally, T-cell interactions with antigen presenting cells (e.g. macrophages, monocytes and dendritic cells) play a significant role in homeostatic conditions and in initiating the adaptive immune response during disease (Mahnke *et al.*, 2008). Furthermore, *in vivo* time lapses indicate that macrophages may preferentially interact with pericytes in a juxtacrine fashion and receive instructions for launching innate immune responses (Stark *et al.*, 2013) and play a role in vascular remodeling (Corliss *et al.*, 2016). Analysis of intercellular colocalization could confirm that macrophages are preferentially migrating to pericytes as part of this process. This method could also be extended to intracellular colocalization studies at high resolutions where imaged structures typically approximated as a point cloud are better approximated as an annular shape. Possible intracellular applications would include characterizing vesicle trafficking across cellular cytoskeletal components (Schuh, 2011).

Although CIRCOAST serves as a new method for hypothesis testing, additional features could enhance its capability. Future work, for example, could include extending the framework to perform power analyses for experiments, along with measuring the effect sizes between groups. Continued research in using a hypergeometric model of random cell placement could yield more accurate results once the distribution’s parameters are better understood for this application. CIRCOAST is also limited to approximating cells as circular shapes, but supporting elongated cell morphologies could facilitate its use in analyzing a greater diversity of cell types and phenotypes. Furthermore, using established methods in characterizing cell morphology (Pincus and Theriot, 2007), simulated cells could be designed to directly represent the heterogeneity in cell size and morphology specific to each image instead of using a single cell shape as an approximation. Currently, CIRCOAST supports the analysis of 2D images that are maximum projections of 3D confocal z-stacks, leading to the issue that cells may appear to colocalize with the projected image but may not be colocalized in the *z*-dimension. Extending CIRCOAST so that it is capable of analyzing 3D image volumes will allow for more accuracy in determining whether cells of interest are colocalizing in the *z*-direction, in addition to the *x*- and *y*-directions. Usage of a nuclear dye would add greater certainty quantifying structures that correspond to distinct cells, especially in the cases where cell clumps form. Indeed, this test could be extended to characterize colocalization of a cell type with itself to study homotypic interactions.

Limitations in generic statistical approaches have been implicated as a contributing factor in the crisis of reproducibility in the biomedical sciences (Nuzzo, 2014). With the development and validation of CIRCOAST, we aim to provide a novel statistical method that is superior to generic hypothesis tests for studying intercellular colocalization, allowing for more robust and repeatable characterization of cell–cell interactions in 2D images of tissues.

## Supplementary Material

Supplementary Material
