# CIRCOAST: a statistical hypothesis test for cellular colocalization with network structures

**DOI:** 10.1093/bioinformatics/bty797

**Published:** 2018-10-04

**Authors:** Bruce A Corliss, H Clifton Ray, James T Patrie, Jennifer Mansour, Sam Kesting, Janice H Park, Gustavo Rohde, Paul A Yates, Kevin A Janes, Shayn M Peirce

*Bioinformatics* (2019) https://doi.org/10.1093/bioinformatics/bty638, 35(3): 506–514.

The publisher wishes to inform the reader that Figures 2, 5 and 6 appeared incorrectly in the above manuscript.

**Fig. 2.**
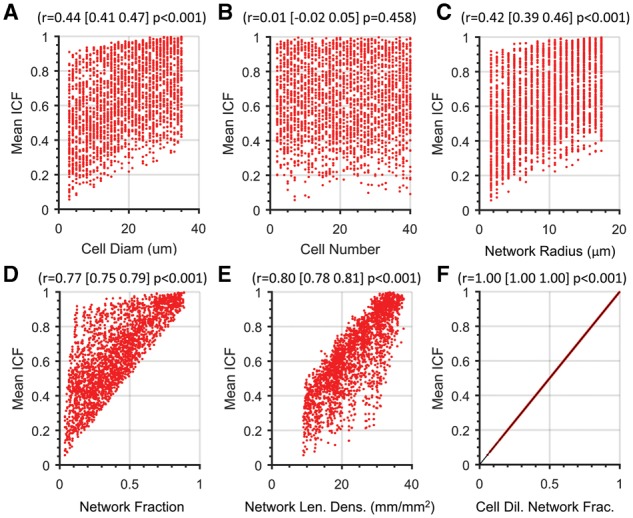
Network area fraction dilated by cell radius determines the random cell colocation fraction. The mean ICF was calculated with the MCMRP over 10 000 trials with randomly selected parameters and displayed as a function of (**A**) cell diameter, (**B**) cell number, (**C**) network radius, (**D**) network fraction, (**E**) network length density and (**F**) cell-dilated network fraction (CDNF, *N*=2 500 images). Pearson correlation coefficient and associated 95% confidence interval and *P*-values are provided at the top of each scatterplot

**Fig. 5.**
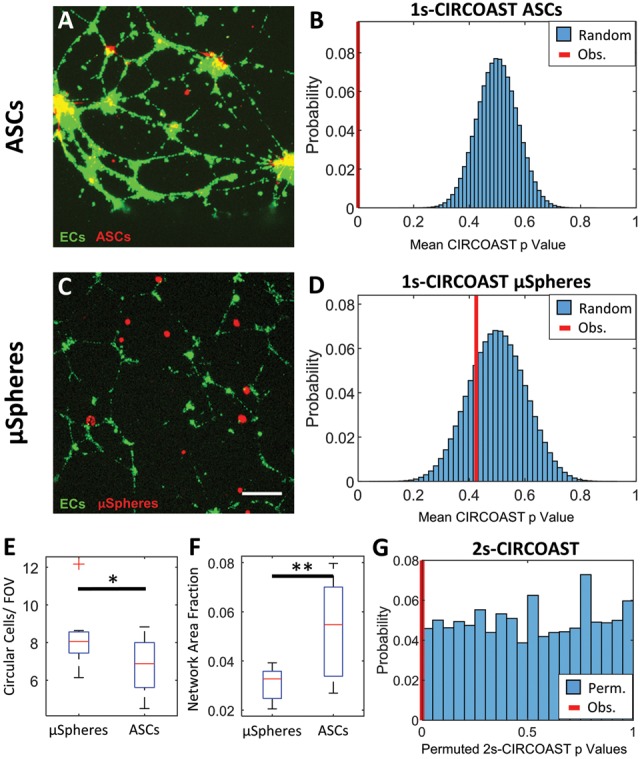
ASCs exhibit enriched colocalization with HUVECS network, while fluorescent microspheres (μSpheres) do not. (**A**) ASCs (red) co-cultured with HUVECS (green). (**B**) Distribution of simulated mean CIRCOAST *P*-values (blue) of random colocalization of ASC group compared to observed mean CIRCOAST *P*-value (red). (**C**) Fluorescent μSpheres seeded on a culture of HUVECs (scale bar 250 um). (**D**) Distribution of simulated mean CIRCOAST *P*-values (blue) of random colocalization from fluorescent μSpheres compared to actual mean *P*-value (red). (**E**) Circular cell density and (**F**) endothelial network density between groups. (**G**) Distribution of *P*-values (blue) derived from permuting CIRCOAST *P*-values in a Wilcox sum rank test between ASCs and μSpheres, with observed *P*-value (red) (*N*=6 wells, 3 images/well)

**Fig. 6.**
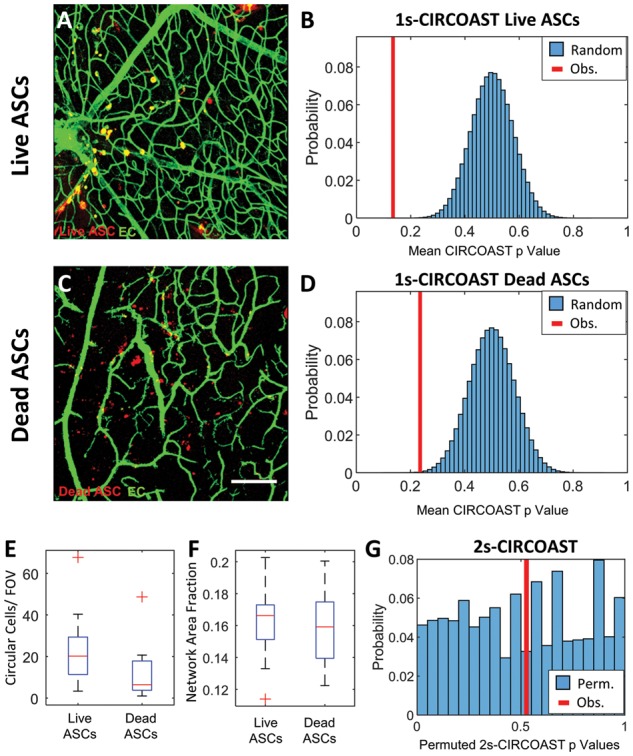
Injected live and dead ASCs both exhibit enriched intercellular colocalization affinity with the vasculature. (**A**) Confocal image of retinal vasculature (green, preprocessed and thresholded) and injected with live DiI-labeled circular ASCs (red). (**B**) Distribution of simulated mean CIRCOAST *P*-values (blue) of random colocalization of ASC group compared to observed mean binomial *P*-value (red). (**C**) Dead DiI-labeled circular ASCs in the retinal vasculature (scale bars 150 um). (**D**) Distribution of simulated mean CIRCOAST *P*-values (blue) of random colocalization from dead cell group, compared to actual mean *P*-value (red). (**E**) Injected circular cell and (**F**) endothelial network density between study groups. (**G**) Distribution of permuted *P*-vales of Wilcox sum rank test of CIRCOAST *P*-values between study groups, with observed *P*-value (red) (*N*=6 mice, 3 images/mouse)

The paper has been corrected online.

