# Supplementary material for: CIRCOAST: a statistical hypothesis test for cellular colocalization with network structures
Source: Bioinformatics. 2018 Jul 19;35(3):506–14. doi: 10.1093/bioinformatics/bty638 (PMC6361237; doi:10.1093/bioinformatics/bty638)
Supplement: Supplementary Material [file bty638_supplementary_material.docx]

CIRCOAST: A Statistical Hypothesis Test for Cellular Colocalization with Network Structures

Bruce A. Corliss^1,ξ,*^, H. Clifton Ray^1,ξ^, James T. Patrie^2^, Jennifer Mansour^3^, Sam Kesting^1^, Janice H. Park^3^, Gustavo Rohde^1^, Paul A. Yates^4^, Kevin A. Janes^1^, and Shayn M. Peirce^1^

^1^Department of Biomedical Engineering, ^2^Department of Public Health Sciences, ^3^Department of Biology, ^4^Department of Ophthalmology, University of Virginia, Charlottesville VA 22902.

*To whom correspondence should be addressed. ^ξ^Equal Contribution.

The Supplementary Material contains –

Supplementary Figures 1 – 12

Supplementary Table 1

Supplementary Notes 1 – 5


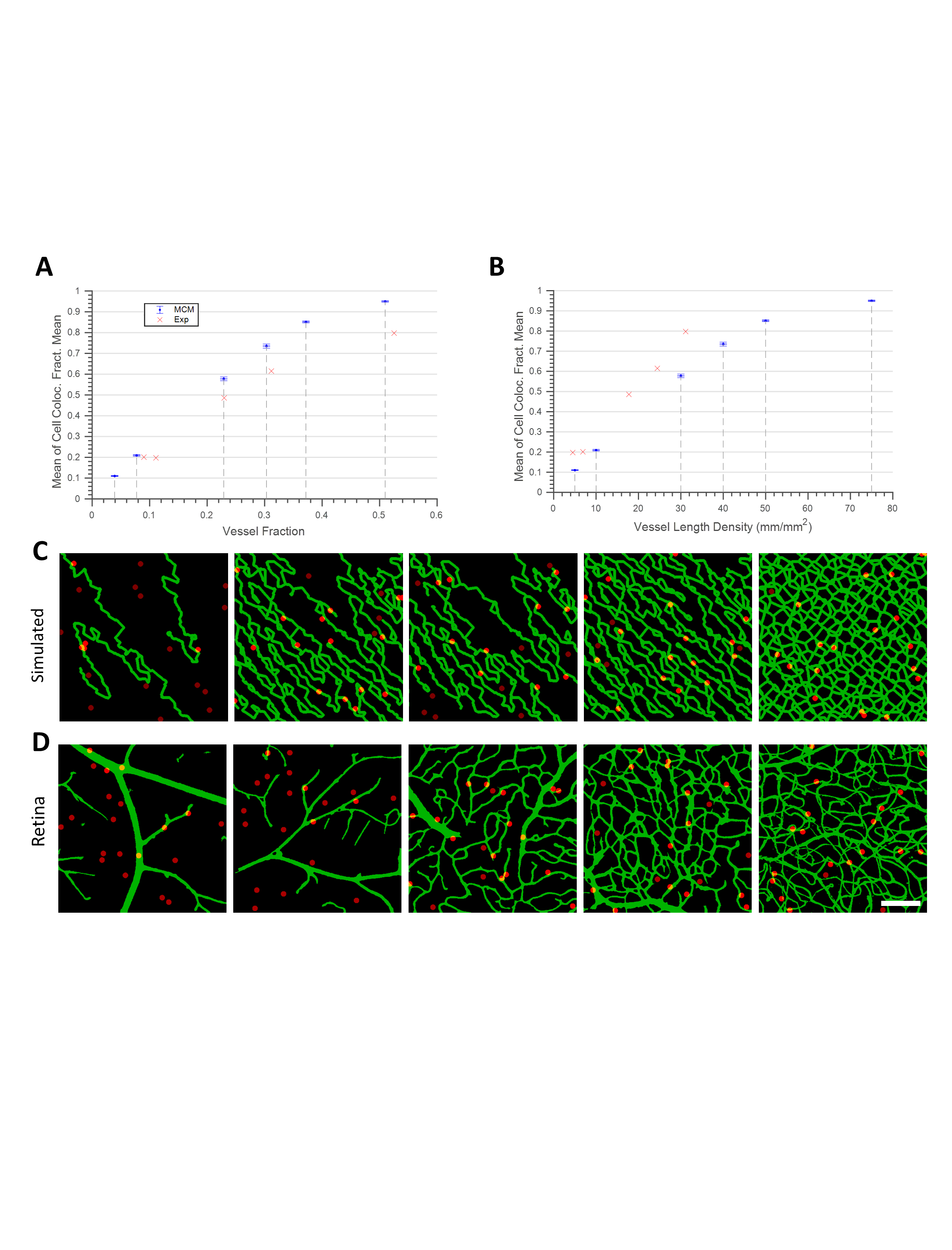


## Supplementary Figures


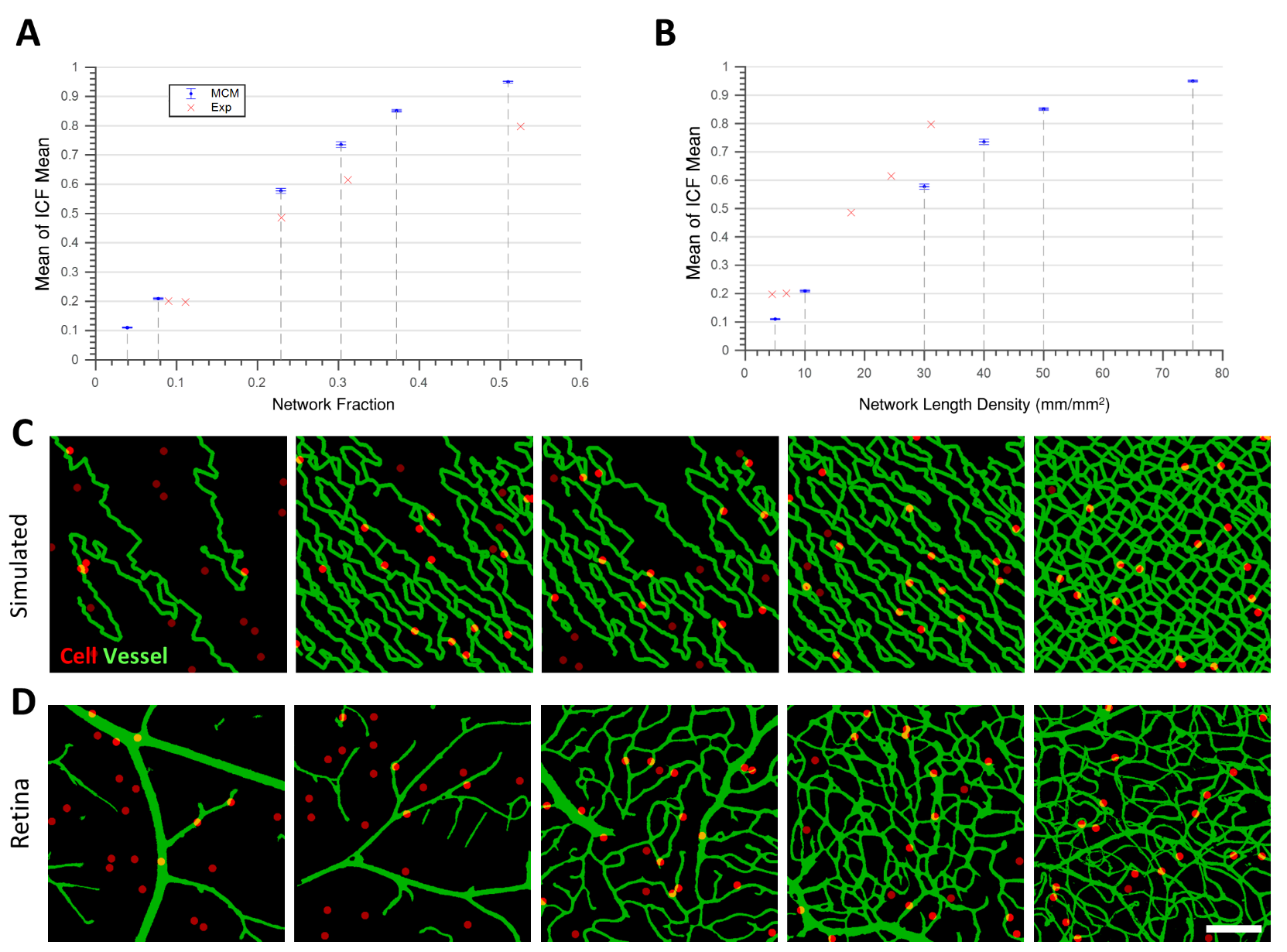


**Supplemental Figure 1: The intercellular colocalization fraction (ICF) with vessel networks due to random chance can be used on a wide range of vessel densities.** **(A, B)** Mean of cell colocation fraction mean versus network length density and network fraction from agent-based Monte Carlo model of random placement (MCMRP) from simulated blood vessel networks (blue) and retinal blood vessels (red, error bars standard deviation). Example images of **(C)** simulated networks (green: vessel) and ones acquired **(D)** in adult murine retina (green: lectin IB4) with simulated cells (red, scale bar 100 µm).


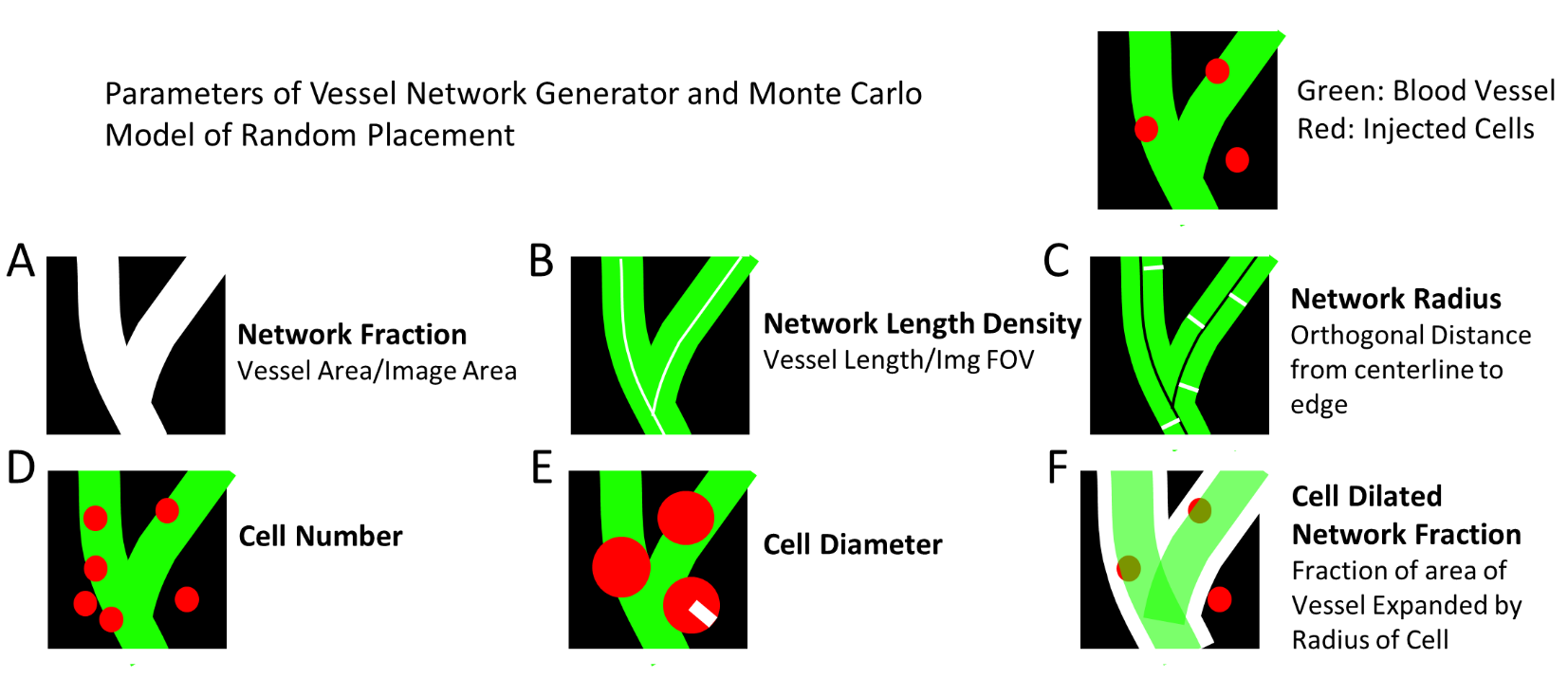


**Supplemental Figure 2. Key Parameters of vessel networks and injected cells. (A)** Network fraction, the fraction of pixels of segmented vessel out of image pixel area. **(B)** Network length density: the length of the centerline of all vessels. **(C)** Network radius: average orthogonal distance from centerline to edge of vessel. **(D)** Cell number. **(E)** Cell diameter. **(F)** Cell dilated network fraction (CDNF): area in which the center of a cell could land and still be colocalized with network (at least 1 pixel overlapping between cell and vessel).


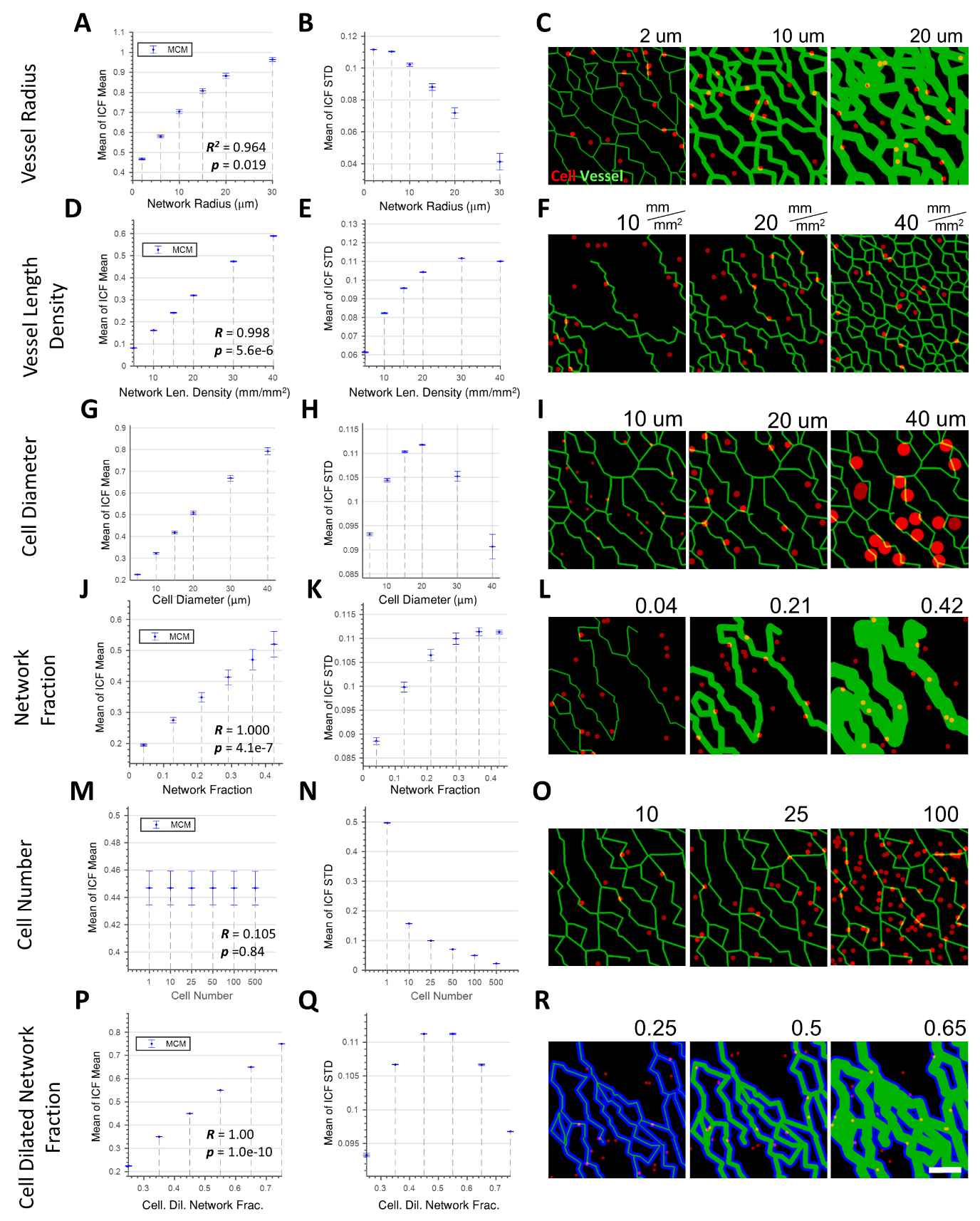


**Supplemental Figure 3: Monte Carlo Model of random placement (MCMRP) reveals all parameters correlate with intercellular colocalization fraction except for cell number.** Mean and standard deviation of mean ICF over sets of images with a single parameter varied, with Pearson correlation of mean ICF (N=8 generated image sets, 10,000 trials/image; red: cells, green: blood vessels). **(A, B)** Mean and standard deviation of mean ICF with varied network radius (network length density 25 mm/mm^2^, cell diameter 15 µm, 20 cells per image), **(C)** with example images labeled by network radius. (**D, E)** Mean and standard deviation of mean ICF with varied network length density (network radius 2.5, cell diameter 15 µm, 20 cells per image), **(F)** with example images labeled by network length density. **(G, H)** Mean and standard deviation of mean ICF with varied cell diameter (network length density 20 mm/mm^2^, network radius 2.5 µm, 20 cells per image), **(I)** with example images labeled by cell diameter. **(J, K)** Mean and standard deviation of mean ICF with varied network fraction (network radius 2.5 µm, cell diameter 15 µm, 20 cells per image), **(L)** with example images labeled by network fraction. **(M, N)** Mean and standard deviation of mean ICF with varied cell number (network length density 20 mm/mm^2^, network radius 2.5 µm, cell diameter 15 µm), with example images labeled by cell number (scale bar 100 µm, error bars are standard deviation).


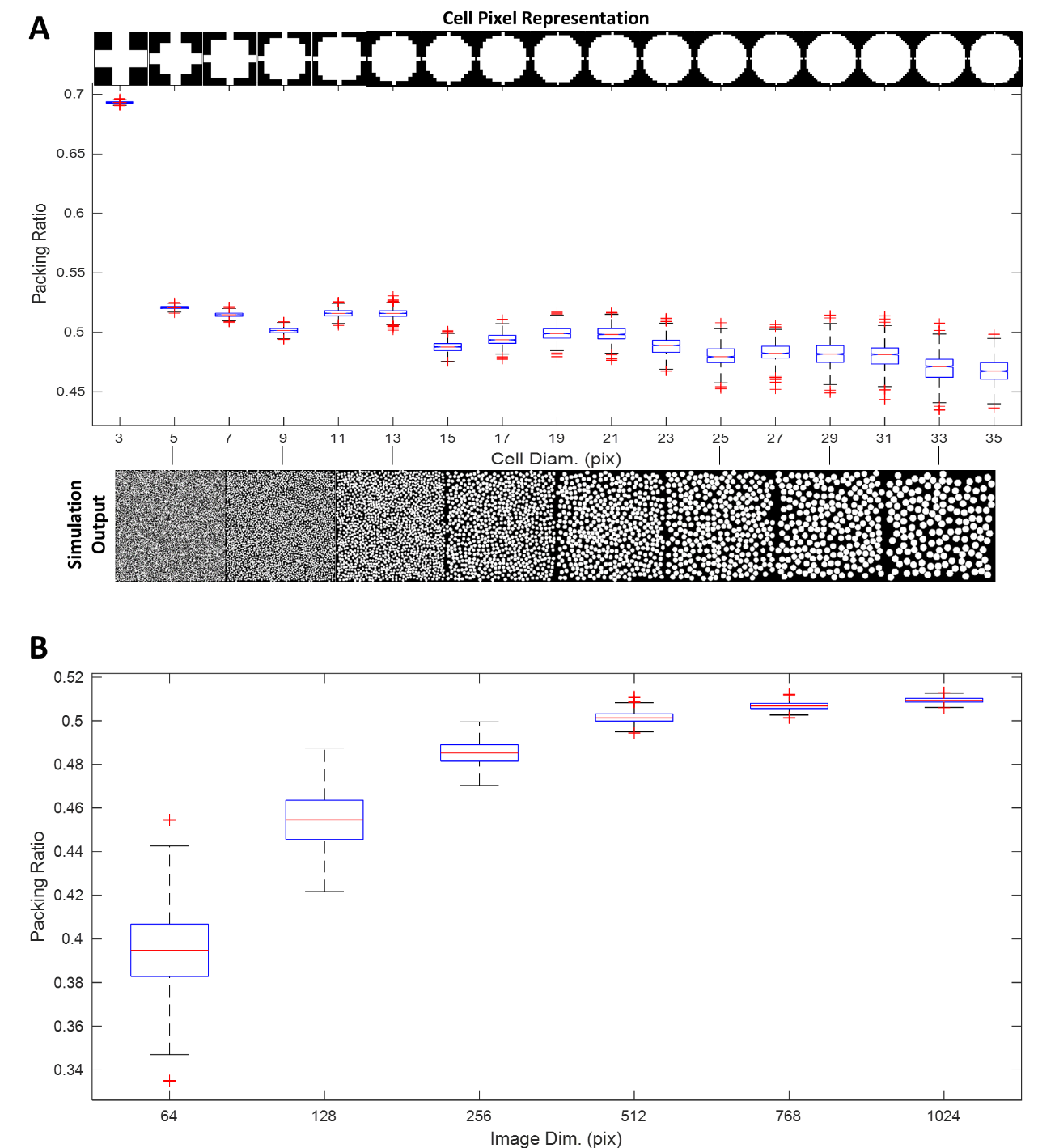


**Supplemental Figure 4: Packing ratio of randomly placed cells changes with cell size and image dimension. (A)** Packing ratio for non-overlapping fully contained randomly placed cells as a function of cell diameter, with example output images and kernel used to represent cells (p=0, Kruskal Wallis, N=100 trials, 512x512 simulated image). Kruskal Wallis used for unequal variances (p=0, Barlett’s Test). For table of packing ratio by cell pixel diameter, see Supplementary Table 1. **(B)** Packing ratio as a function of image pixel dimension (p=0, Kruskal Wallis, N=100 trials, cell diameter 9 pixels). Kruskal Wallis used for unequal variances (p=0, Barlett’s Test).


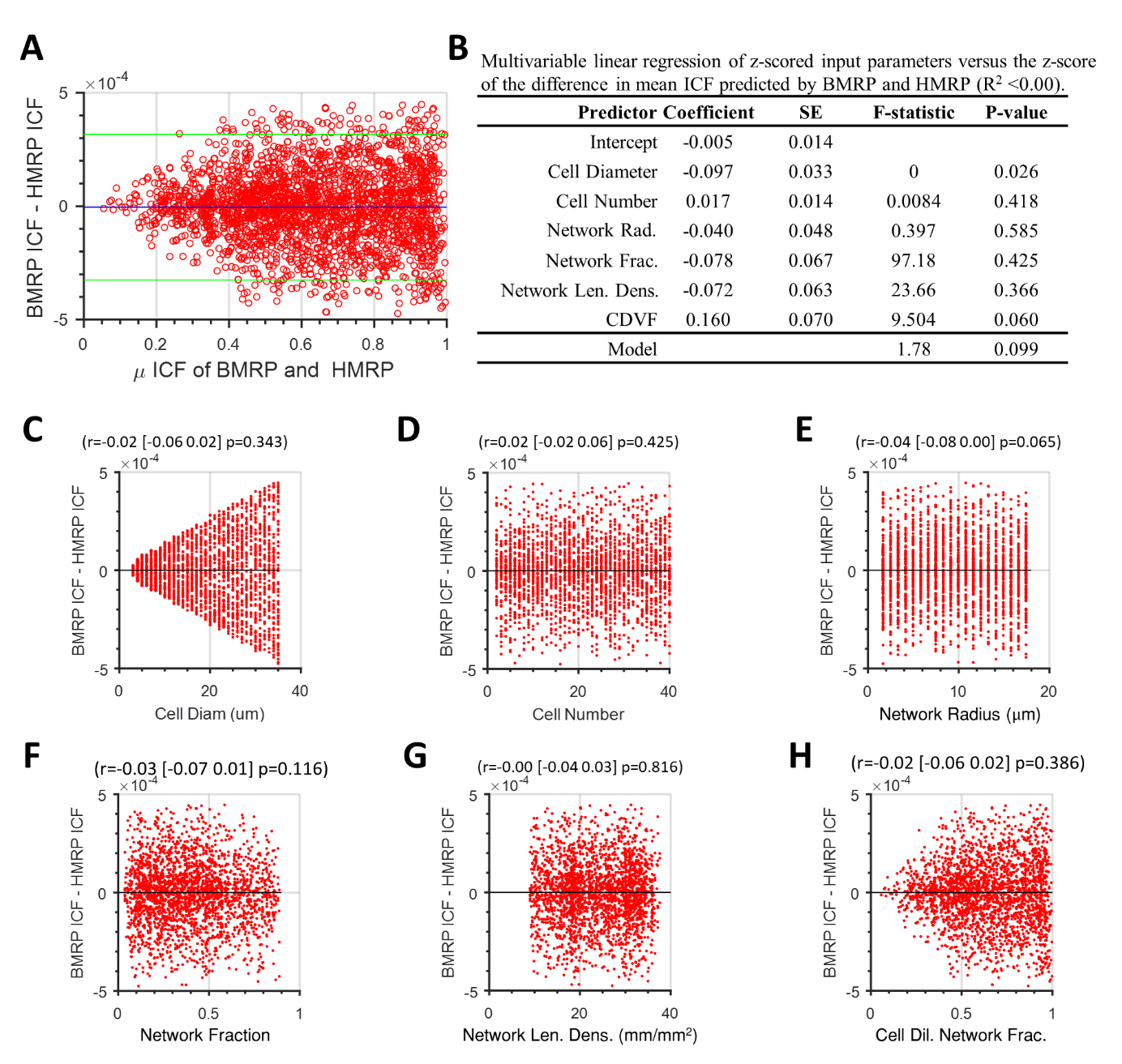


**Supplementary Figure 5:** **Discrepancy between BMRP mean ICF and HMRP mean ICF versus the cell and vessel network input parameter values.** **(A)** Bland Altman plot showing disagreement between models. **(B)** Multi-variate linear regression of z-scored predictors versus z-scored difference between models **(C-H)** Pearson correlation of each predictor versus difference between BMRP and MCMRP (Pearson r with [95 % CI] and p value).


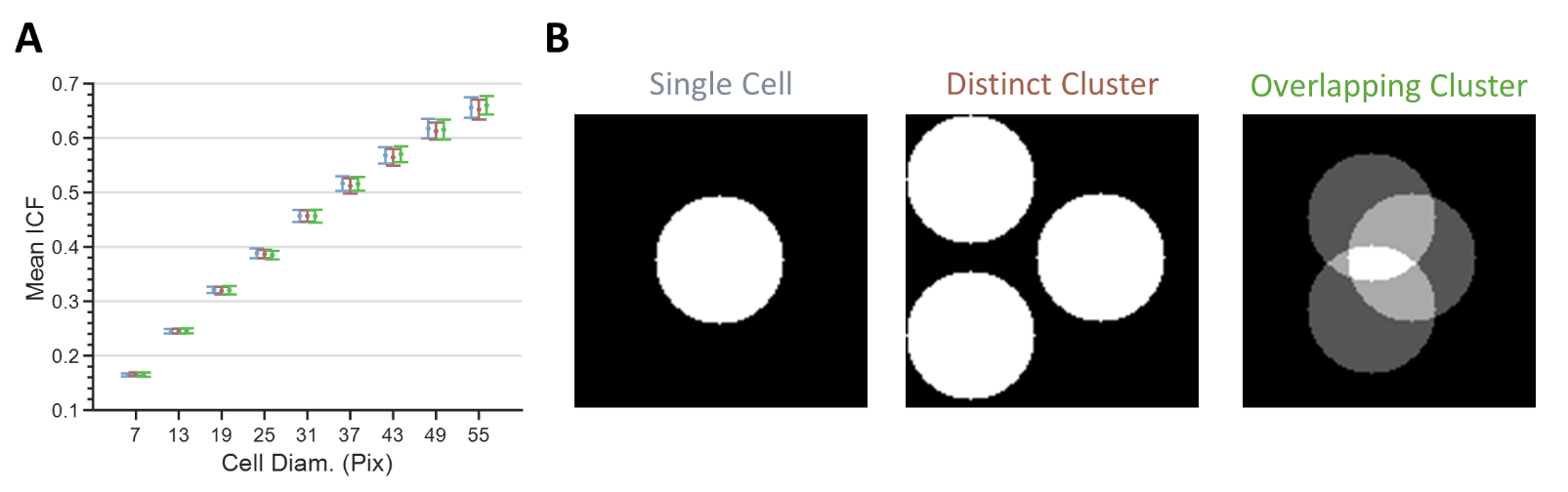


**Figure 6: Cells randomly placed in clusters yields no difference in ICF as randomly placed individual cells. (A)** Plot of mean ICF from random cell placement (BMRP) over a range of cell diameters with either random placement of a single cell (Single Cell), random placement of a non-overlapping cluster of three cells (Distinct Cluster), or random placement of a cluster of three overlapping cells (Overlapping Cluster) (p=0.996 for cell placement method, 2-way ANOVA, error bars are 95% confidence interval of the mean, N=10 simulated vessel images with network length density of 15 mm/mm^2^, 1000 simulations per data point). Each study group had the same total number of cells per trial in simulations ran, whether placed individually or in clusters. **(B)** Example images of cell kernels for each of the cell placement methods (21 cells placed in an image with pixel dimensions of 512 by 512, single vessel image used across all data points).


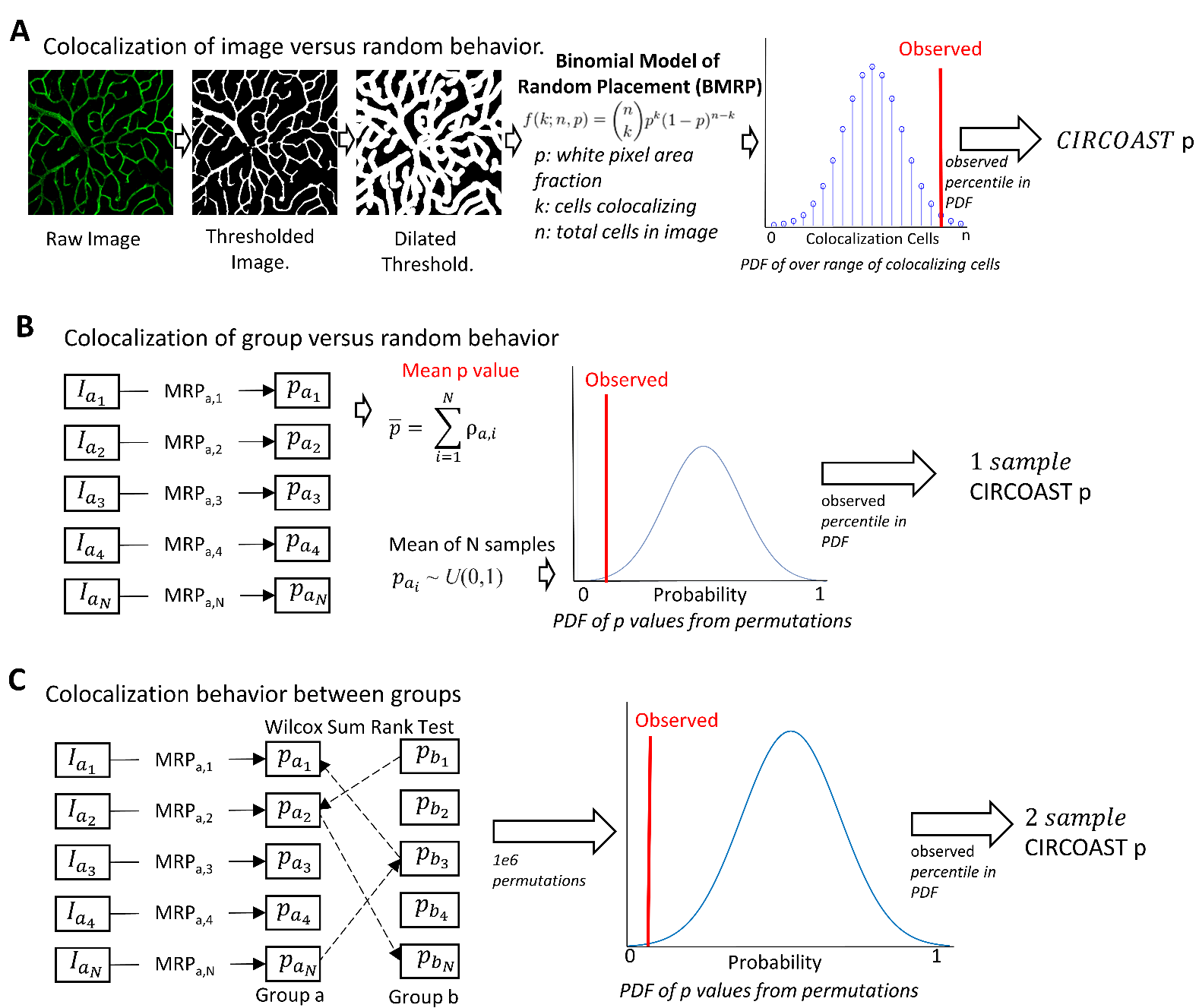


**Supplemental Figure 7: CIRCOAST process pipeline for testing for non-random and unique colocalization between study groups. (A)** The process to determine if a single image exhibits enriched colocalization, where the observed number of colocalizing cells is compared against a PDF generated by a model of random placement (MRP) to obtain a p value (either MCMRP, BMRP, or HMRP). **(B)** Process to determine if images from a study group exhibits enriched colocation over random behavior, where the mean of all the CIRCOAST p values from the images is compared to a PDF generated by p values assuming random behavior (sampled from a uniform PDF) to generate a 1-sample CIRCOAST p value. **(C)** Process to determine if colocation behavior using two study groups differs by comparing Wilcox Sum Rank Test of CIRCOAST p values compared to a PDF of WSRT of permuted samples to generate a 2-sample CIRCOAST p value.


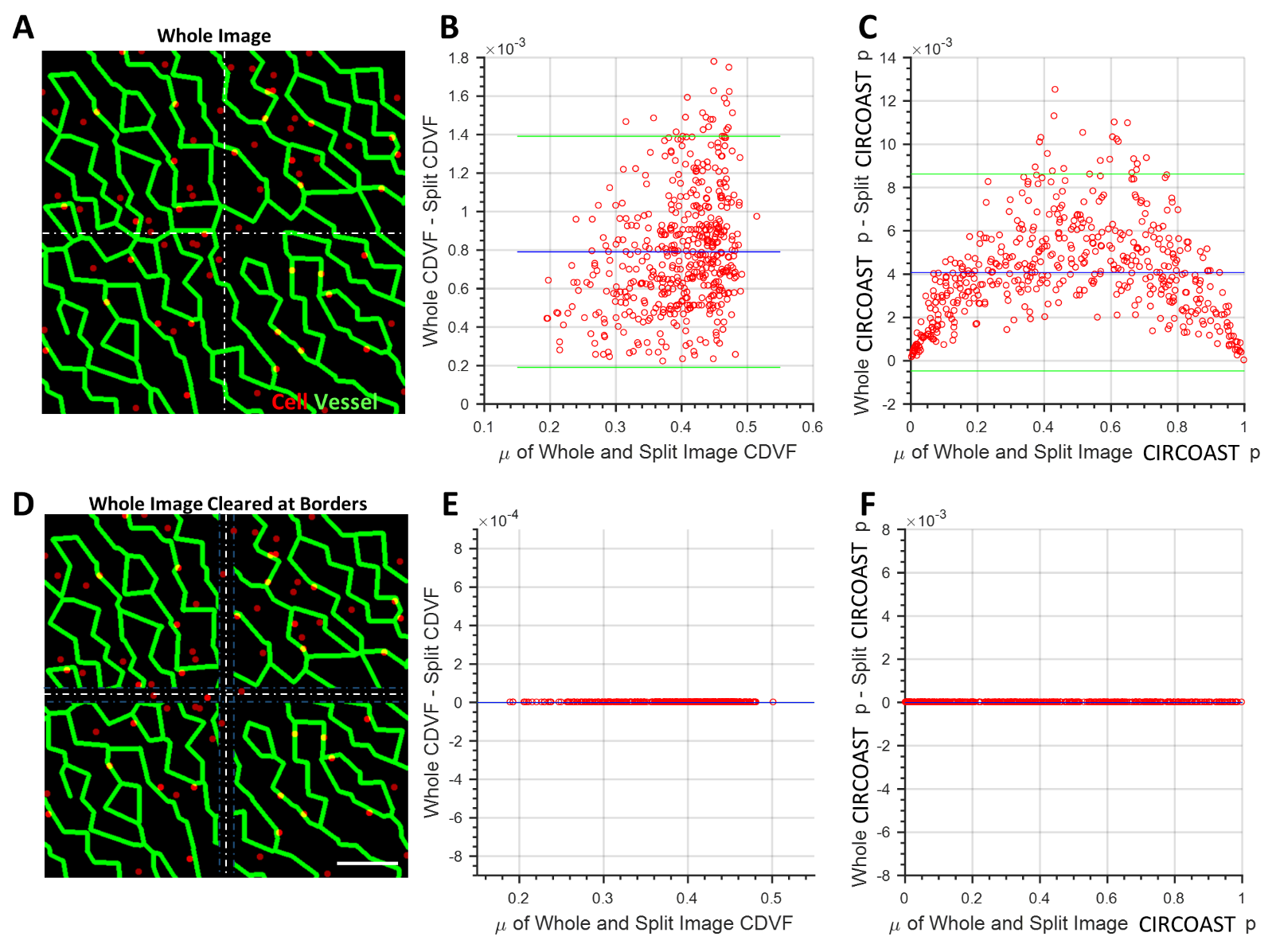


**Supplemental Figure 8: Calculating the combined CDVF and summing injected cells across images yields the same CDVF and CIRCOAST p from an image before and after splitting**. **(A)** A dataset of simulated vasculature and injected cells were generated, and the CDVF and CIRCOAST p value calculated for each image split into fourths (white line) compared to the original image, **(B)** revealing a small discrepancy in CDVF (p=6.60e-155, paired t-test) and **(C)** CIRCOAST p (p=4.75e-223, paired t-test) in split images versus whole. This disagreement was hypothesized to be from border effects of the split images, meaning the cell dilated vessel area from one split image could land across the image border to the adjacent image (an artifact of this validation test). **(D)** When the vasculature was removed one cell distance from the image border (white line) between sub images, the discrepancy no longer existed with **(E)** CDVF (p=NaN, paired t-test, values exactly the same) and **(F)** CIRCOAST p (p=NaN, paired t-test, values exactly the same) in the split images versus whole, indicating that this method is valid for combining information between images from a single biological replicate. N=500 images generated with vessel length density 29.4 ± 5.1 mm/mm^2^ and 80 injected cells per field of view, with 8 µm cell size, scale bar 100 µm.


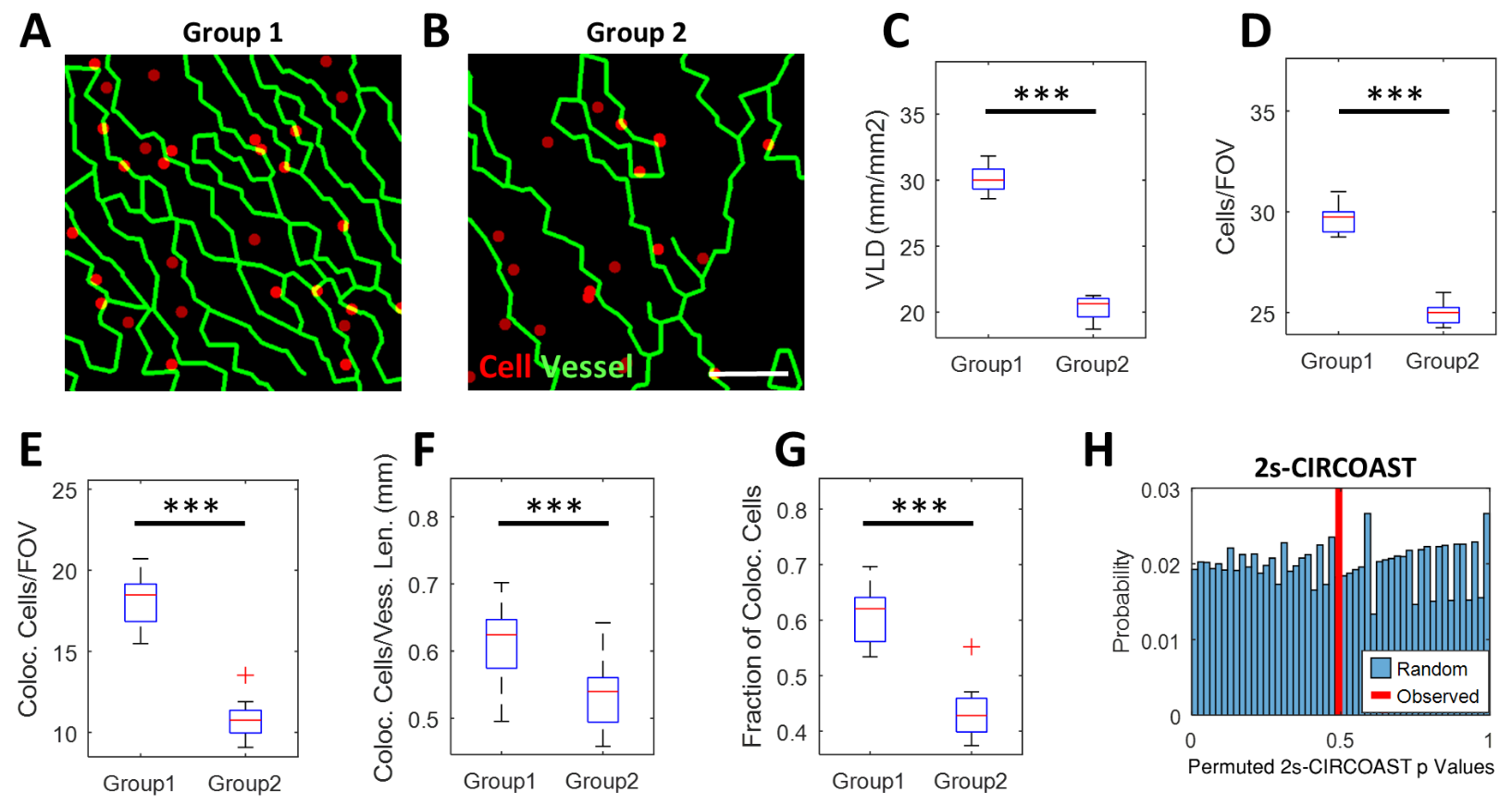


**Supplemental Figure 9: Generic statistics leads to incorrect conclusions with ICF when confounded with cell and vascular density changes**. A dataset of two study groups were generated, **(A)** one with high vascular and circular cell density (Group 1), **(B)** and one with lower vascular and circular cell density (Group 2) to represent the dropout seen in diabetes. Using the vessel network generator and Monte Carlo model (N=10 samples per group, n = 4 images per sample), cells were randomly placed in vascular images with a uniform distribution. A valid statistical method should reveal no changes in cellular colocalization behavior between groups. **(C)** Vessel length density decreased 33% (p=2.05e-14, 2 sample t-test) from group 1 (30.13±1.04 mm/mm2) to group 2 (20.24±0.98 mm/mm2). **(D)** Total cell density per field of view decreased 16% (p=2.07e-12) from group 1 (29.80±0.77cells) to group 2 (24.95±0.50 cells). **(E)** Examining the data by measuring colocalizing cells per FOV led to an erroneous conclusion that there was a 41% decrease in colocalization behavior (p=2.46e-09, 2 sample t-test) from group 1 (18.27±1.71cells) to group 2 (10.83±1.33 cells). **(F)** Examining the data by measuring colocalizing cells per vessel length (mm) led to an erroneous conclusion that there was a 12% decrease in colocalization behavior (p=1.12e-02, 2 sample t-test) from group 1 (0.61±0.06 cells per 1 mm vessel) to group 2 (0.54±0.05 cells per 1 mm vessel). **(G)** Examining the data by measuring the fraction of colocalizing cells led to the erroneous conclusion that there was a 30% decrease in colocalization behavior (p=6.68e-07, 2 sample t-test) from group 1 (0.61±0.05 cell fraction) to group 2 (0.43±0.05 cell fraction). **(H)** However, using the permuted binomial model of random placement for the cellular colocalization affinity with vasculature test, no change in colocalization behavior was correctly observed between groups (p=0.494 2-sample CIRCOAST, permuted WSRT, 1e7 permutations).


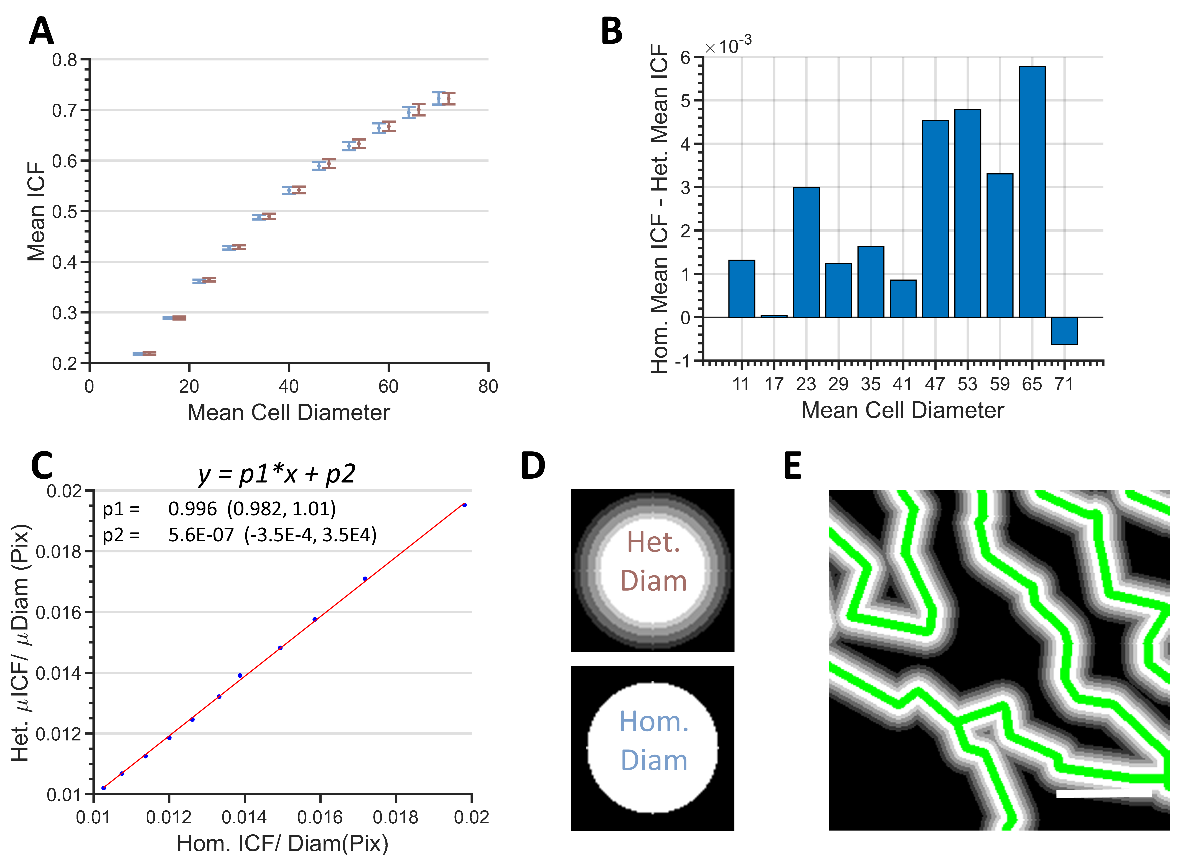


**Supplemental Figure 10: Randomly placed cell populations of heterogeneous diameter yields similar ICF as cells with uniform mean diameter**. **(A)** Comparison of mean ICF between random placement with the BMRP of homogenous cell size (blue) versus a heterogeneous mix of cell diameters (brown) around the homogenous value, showing a difference in mean ICF (p=0.038, paired t-test), but **(B)** very small effect size between groups (cell diameter sampled with uniform distribution from 90%, 95%, 100%, 105%, and 110% from mean, N=10 simulated vascular images with vessel length density of 15 mm/mm^2^, 1000 simulations run per data point, error bars are 95% confidence error of the mean). **(C)** Since the mean of the sampled heterogeneous diameters do not exactly correspond to the homogenous cell diameter, ICF of heterogeneous group is normalized by mean of the sampled diameters (from the simulations run for each data point) compared to ICF of homogenous group normalized by diameter, with a fitted line whose 95% confidence interval (brackets) includes a value of one, suggesting a one to one correspondence between axes. **(D)** Example cell kernels used, with shades of gray in the heterogeneous diameter group denoting different size kernels. **(E)** Example vascular network image (green) with CDNF from a single set of cell kernels from the heterogeneous group (shades of grey, scale bar 25 um).


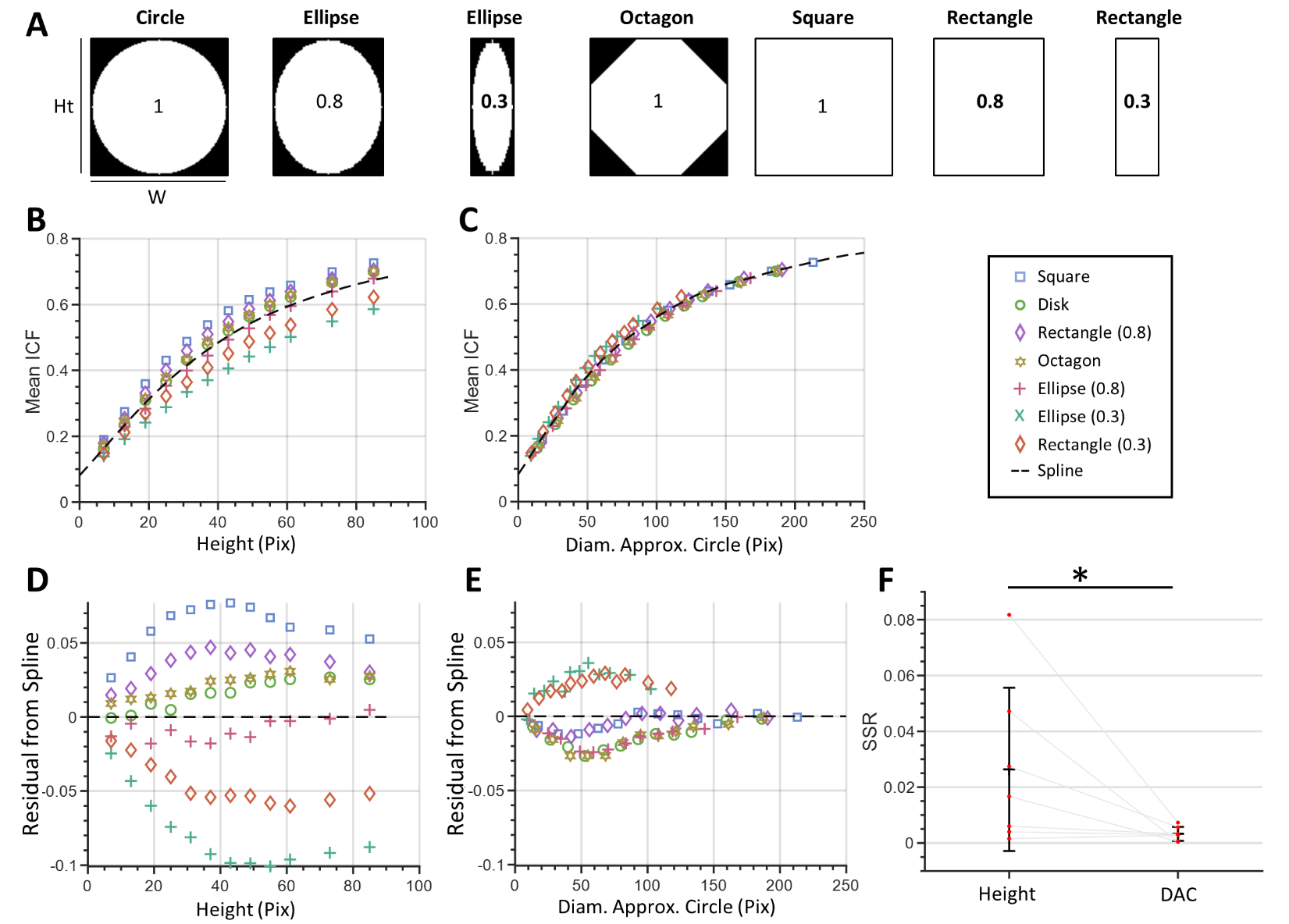


**Supplemental Figure 11: Randomly placed geometric shapes have closer agreement with ICF when characterized by diameter-approximated circles (DAC) compared to cell height.** **(A)** Library of kernels used to characterize how cell shape influences ICF from random kernel placement, with numeric values denoting the ratio between width and height. Kernels were characterized by either their height or the mean of their diameter-approximated circle (DAC), defined as the diameter of a circle whose area is equal to the area of the evaluated shape. The mean ICF from random placement was calculated using the BMRP for all shapes across a range of kernel sizes and plotted by both **(B)** Height and **(C)** DAC, with a smoothing spline fitted across all points (smoothing parameter 0.005 in MATLAB fit() function, N=10 simulated vascular images with vessel length density of 15 mm/mm^2^, 1000 simulation runs per datapoint). The residuals of all shapes relative to the fitted smoothing spline by **(D)** Height and **(E)** DCA. **(F)** Sum of squared residuals (SSR) for the ICF from each of the shape groups compared to fitted smoothing spline, with an 82% reduction in mean SSR of DAC compared to Height (p= 0.0469, paired Wilcoxon signed rank test, N=7 shape types), and a 99.3% reduction in the variance of SSR of the DAC compared to Height (p=0.0157, paired permuted F-test, 1e6 permutations). (Data in (F) determined non-normal by One-sample Kolmogorov-Smirnov test: Height p=5.9E-13, DCA p=1.16E-8).

**
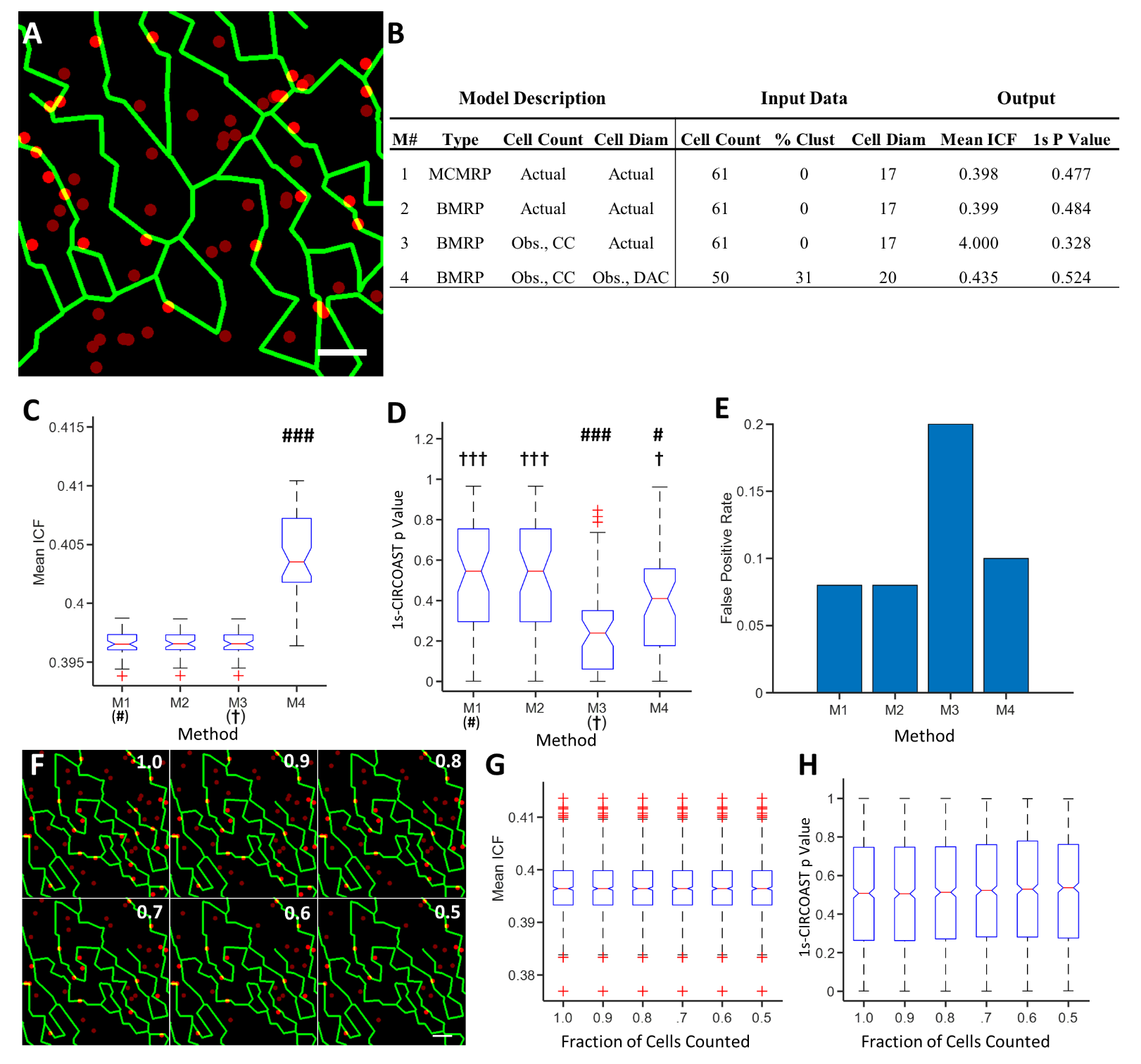
**

**Supplement Figure 12: Variability in image analysis can change p values of CIRCOAST test**. In order to investigate the effect of flawed input data on falsely altering the outcome of the CIRCOAST test, (**A**) a dataset of 2000 images were created with homogenous vessel length density (19.6 ± 0.12 mm/mm^2^) and randomly seeded elevated total cell counts (60.4 ± 2.0 cells) to cause very significant overlap events to mimic a worst-case scenario of heavily flawed input data quantification (21.2% ± 1.1 cells part of multicellular clusters and misrepresented) (scale bar 100 um). Images were split randomly into 50 study groups with 20 images/ study group. (**B**) Four methods of analyzing the input data were used, with example output from a single image shown with the table: (Method 1: idealized) counting cells based on their coordinate (so all cells are counted correctly regardless of degree of overlap) and actual cell diameter using the MCMRP, (Method 2: idealized) counting cells based on their coordinate and actual cell diameter using the BMRP, (Method 3: flawed) cell counting based purely on connected components (worse case for cell counting, if cells touch they are considered one cell) and actual cell diameter using the BMRP, (Method 4: flawed with mitigation) cell counting based purely on connected components and cell diameter calculated using the diameter approximated circle method (DAC) from mean connected components area using BMRP. (**C**) The mean ICF across all images in a study group was compared between methods (p=5.95e-54, 1 way ANOVA), along with (**D**) 1-sampe CIRCOAST p value for each study group (p=1.31e-07, 1 way ANOVA), and (**E**) false positive rate across study groups (# denotes significance from Method 1, † significance from Method 3). To model the effect of cell counting with missing cells in input images, (**F**) a portion of the cells in the input images were counted (100, 90%, 80%, 70%, 60%, 50%), and (**G**) mean ICF predicted by BMRP (p=1.0, 1 way ANOVA, N=2000 images/ study group) and (**H**) 1S CIRCOAST p values compared between groups (p=0.540, 1 way ANOVA, N=2000 images/ study group, tukey-kramer multiple comparisons) (scale bar 100 um).

## Supplementary Table

### Supplementary Table 1: Packing ratios of randomly placed non overlapping cells as a function of cell pixel diameter.

| Cell Diam. (Pix) | Mean Pack. Ratio | STD Pack. Ratio |  | Cell Diam. (Pix) | Mean Pack. Ratio | STD Pack. Ratio |
| --- | --- | --- | --- | --- | --- | --- |
| 3 | 0.69325592 | 0.000914904 |  | 31 | 0.48027837 | 0.010227747 |
| 5 | 0.520709251 | 0.001339616 |  | 33 | 0.47099324 | 0.011287194 |
| 7 | 0.514762058 | 0.0019614 |  | 35 | 0.46812859 | 0.010955898 |
| 9 | 0.50140456 | 0.002446035 |  | 37 | 0.465189861 | 0.012521895 |
| 11 | 0.515966206 | 0.003274793 |  | 39 | 0.463105167 | 0.013354375 |
| 13 | 0.515891399 | 0.003867109 |  | 41 | 0.460029854 | 0.013564348 |
| 15 | 0.487618847 | 0.00450094 |  | 43 | 0.452998238 | 0.014227795 |
| 17 | 0.493858704 | 0.005078989 |  | 45 | 0.451910263 | 0.015901951 |
| 19 | 0.498641937 | 0.005734494 |  | 47 | 0.448864563 | 0.015581893 |
| 21 | 0.499130131 | 0.006595696 |  | 49 | 0.442819237 | 0.016561947 |
| 23 | 0.48872485 | 0.007417942 |  | 51 | 0.441543674 | 0.017762362 |
| 25 | 0.480217346 | 0.007926075 |  | 53 | 0.437041992 | 0.01872689 |
| 27 | 0.483155647 | 0.008468317 |  | 55 | 0.434697292 | 0.019495479 |
| 29 | 0.481239983 | 0.009415811 |  |  |  |  |

Note: Image dimension: [512 512], N=100 trials per cell diameter.

## Supplementary Notes

### Supplementary Note 1: Monte Carlo Algorithm

The Monte Carlo simulation requires a binary image with vasculature in the image foreground (white pixels). For images that have high signal-to-noise ratio (SNR) for the vasculature, simple thresholding controls are provided within the GUI so the user can produce a binary vasculature image with minimal effort. Alternatively, for images that are more difficult to analyze due to low SNR, uneven background signal, or low contrast, the user can process these images with specialized image processing routines using an external program such as ImageJ, Photoshop, MATLAB, or Python, export the binary, and import it into CIRCOAST.

When the user first imports an image, the program displays the image data and determines whether it is a binary. If the image is not binary, it is converted into an 8-bit RGB and the user has the option to activate thresholding on one or multiple of the three channels with the controls on the right side of the program window. The vertical slide bars are used by the user to select a relative threshold for that channel that is scaled to the image’s maximum pixel value. The threshold is computed and displayed in real time as the settings are adjusted. With the default behavior, any pixel greater than the threshold will become a white foreground pixel, but this can be inverted for each channel independently. If thresholding is completed on multiple channels, the resulting binary image is the union of the results from each channel.

Once a proper binary image of the vasculature is produced, the user sets the resolution of the image, the average diameter of the injected cell, the total number of injected cells found in that particular field of view, and the total number of trials run for the simulation. To initialize the Monte Carlo simulation, the user runs a single trial and examines the simulated cell size in the output image to verify that it qualitatively resembles those observed experimentally. This first trial also allows the program to provide a rough conservative estimation of the execution time for the entire simulation, and also calculate how many trials can be calculated concurrently based on the amount of memory that is available to MATLAB for that system. When the full simulation is run, the program runs trials in batches, trading system RAM for accelerated execution speed, and provides updates with progress and time to completion.

The segmented vasculature undergoes a Euclidean distance transform to yield an image where each black pixel contains the distance to the closest white foreground pixel (vasculature). Sets of pixel coordinates that represent the locations of cells from a single trial are used to calculate the distance of each cell to the vasculature by sampling pixel values of the Euclidean distance transformed image of the vasculature. The distance values obtained from each cell are thresholded by the radius of the injected cell: cell distances less than or equal to the injected cell radius are considered colocalizing with the vasculature. The fraction of cells colocalizing is calculated for each trial, and the process is repeated until all of requested trials for the simulation is complete. The mean and standard deviation of the intercellular colocalization fraction is calculated across all trials and displayed as output.

### Supplementary Note 2: Algorithm for Vessel Network Generator

The vessel network generator can create vessel networks with finely tuned parameters in a stochastic fashion. A point cloud is generated that marks the location of avascular areas in the network, and vessel segments are generated by applying a watershed, bisecting all points and creating a fully connected network with a honeycomb appearance. Points are iteratively added until a fully connected target vessel length density (VLD) or vessel area fraction (VAF) is reached, and then vessel segments are stochastically removed until a target final VLD or VAF is reached. Segments are removed according to a set of rules to reduce the density and interconnectedness of the network while maintaining the appearance of a vascular bed found in vivo.

To generate the point cloud used to seed the watershed segmentation, locations in a binary image are marked iteratively with a uniform probability distribution. To avoid populating points too closely, the image indices around each point added are removed from the list of possible spawn locations for future points. After each point is added, a watershed is computed on the image and the VLD and VAF is calculated. If these values exceed the target values for the initial fully connected network, then the process of adding spawn point ceases, otherwise it continues until this condition is satisfied or no available spawn points are left in the image.

Once the vascular network has been populated, line segments are then iteratively removed and VLD and VAF is calculated until the final target value is reached. Line segments are prioritized with a scoring system that factors in segment orientation, line segment length, hole area that segments border, and whether the segment connects to an endpoint in the network. Additionally, the score is influenced with random probability to provide a stochastic element to the regression of vessel networks. The segments with the highest score is removed, and then all segments are rescored for each iterative round.

### Supplementary Note 3: Cell Sources

**Cell Sources**: Human umbilical vein endothelial cells (HUVECS) were generously donated by Dr. Brian P. Helmke (University of Virginia). Mouse adipose-derived stem cells (mASCs) were obtained by adapting the previously described protocol (Mendel et al. 2013; Cronk et al. 2015 Mar 13). Briefly, epididymal fat pads of male mice were digested in type I collagenase digestion buffer for 1 h at 37°C. Digested tissue was filtered through a 200-µm mesh to discard undigested tissue, and excess collagenase was removed through centrifugation. Collected cells from centrifugation were incubated in red blood cell lysis buffer (eBioscience) for 5 min to remove red blood cells. The remaining cells were filtered through a 70-µm filter, and plated on tissue cultured treated plastic.

Isolated HUVECs were cultured on tissue culture treated plastic (Corning) in Endothelial Basal Medium-2 (Cat. no. CC-3156, Lonza supplemented with Endothelial Cell Growth Medium-2 SingleQuot Kit Supplements and Growth Factors (Cat. no. CC-4176, Lonza, Walkersville, MD). mASCs were cultured on tissue culture treated plastic (Corning) in Gibco DMEM/F12 supplemented with 10% FBS and 1% Antibiotic-Antimycotic. Cells were passaged once they were 80% confluent using Stempro® Accutase® (Thermo). HUVECS were used from passage 7 to 10 in all studies, and mASCs were used from passage 4 to passage 6 in all studies. All cells were incubated at 37°C, 5% CO2, and 95% humidity.

### Supplementary Note 4: In Vitro and In Vivo Validation

**In Vitro validation**: 200 µL of Cultrex® Basement Membrane Extract (BME) Reduced Growth Factor (Cat. no. 3433-005-01, Trevigen, Gaithersburg, MD) was added to the single wells of 8-well Nunc™ Lab-Tek® II Chamber slides (Cat. no.154534, Thermo Scientific, Waltham, MA), and the chamber slides were incubated at 37°C to solidify the BME. Prior to seeding in BME, HUVECs were labeled with Vybrant® DiO Cell-Labeling Solution (Cat. no. V22886, Thermo Scientific), and mASCs were labeled with Vybrant® DiI Cell-Labeling Solution (Cat. no. V22885, Thermo). A total of 40,000 HUVECs were added to each individual well in the chamber slides that contained BME. Immediately after seeding the HUVECs, 400 DiI-labeled mASCs, and/or FluoSpheres® Polystyrene Microspheres (Cat. no. F21012/F8843, Thermo) were immediately added to each individual well. 24 h after seeding the appropriate cells and microspheres in the BME, the network assays were imaged using a Leica TCS SP2 confocal with a DMIRE2 inverted micro-scope to determine the colocalization of microspheres and mASCs to the HUVEC network.

**In Vivo Validation**: All procedures performed with mice conformed to the guidelines within the ARVO Statement for the Use of Animals in Ophthalmology and Vision Research and were approved by the University of Virginia’s Animal Care and Use Committee. Using previous techniques (Mendel et al. 2013), C57Bl/6J mice were immersed in 75% O2 from postnatal day 7 (P7) to postnatal day 12 (P12). At postnatal day 12, mice were returned to normoxia and 10,000 viable mASCs in 1.5 μL of PBS were injected into the vitreous gel of the eye. In the contralateral eye, 10,000 dead mASCs were injected in 1.5 μL of PBS. Dead cells were exposed to 4% PFA for 10 minutes and then washed with PBS. All cells were labeled with Vybrant® DiI Cell-Labeling Solution (Thermo) prior to intravitreal injections. Mice were euthanized 4 weeks post-intravitreal injections, and the eyes were harvested and fixed in 4% PFA for 10 min. Retinas were then dissected from each eye, flat-mounted on coverslip glass, and permeabilized with 1 mg/mL digitonin. Retinas were stained with isolectin GS-IB4 Alexa Fluor 647 Conjugate (Cat. no. I32450, Thermo) and SYTOX® Green Nucleic Acid Stain (Cat. No. S7020, Thermo). Retinas were imaged using a Leica TCS SP2 confocal with a DMIRE2 inverted microscope to determine the colocalization of micro-spheres and mASCs to the HUVEC network.

### Supplementary Note 5: Image Acquisition, Thresholding, and Quantification

**Image Acquisition**: All biological samples were imaged on a Leica TCS SP2 confocal with Nyquist sampling. Retina samples were imaged with a 20X (0.7 NA, air, HC PL APO) objective lens and a confocal pin size of 2.38 AU, where a 20 µm z-directional volume was obtained at a sampling rate of 2 µm per optical z-slice. Frame-averaging was set at 2 and images were collected at a scan speed of 400 Hz. Pixel Resolution was set at 1024x1024 resulting in an image resolution of 0.791 μm/pix. Sytox Green and isolectin IB4 was imaged sequentially by laser excitation of 458 nm and 633 nm, respectively. For this laser excitation, emission bandpass filters were set at 470-55 nm and 650-750 nm to collect the fluorescent signal. DiI was imaged at a laser excitation at 561 nm and the fluorescent signal was collected at an emission bandwidth of 575-625 nm.

Similarly, cell and bead cultures were imaged using a 10X (0.3 NA, air, HC PL FLUOTAR) objective lens with a confocal pin size of 2.38 AU, where a 300 µm z-directional volume was obtained at a sampling rate of 16 µm per optical z-slice. Frame-averaging was set at 2 and images were collected at a scan speed of 400 Hz. Pixel resolution was set at 1024x1024 resulting in an image resolution of 1.56 μm/pix. DiO and FluoSpheres™ Polystyrene Microspheres 645/680 (Thermo) were imaged concurrently by laser excitation of 488 nm and 633 nm, respectively. Emission bandpass filters were set at 500-550 nm and 650-750 nm to collect the fluorescent signals. DiI was imaged at a laser excitation at 561 nm and the fluorescent signal was collected at an emission bandwidth of 575-625 nm.

**Image Acquisition Rationale**: It is recommended that z stack sampling be as close to Nyquist sampling as is practical to capture all cell structures that could define colocalization events. Image acquisition parameters and z-stack volume should be kept constant between study groups to minimize any bias that the imaging could introduce. In terms of how much volume should be covered in a z-stack, it should large enough to capture the vessel network and enough COIs, but the larger the volume the greater chance for false positives from overlap between objects that are not actually colcoalized in the z axis.

**Image Thresholding**: The DiO channel that labeled endothelial cells in the in co-culture experiment was processed by thresholding with an adaptive median-filtered background subtraction. In short, the DII channel was blurred with a median filter kernel 200 pixels in size, which was subtracted from the original image to create the foreground image. The foreground image was thresholded for values greater than 40 to be considered to be pixels marking endothelial cells. The segmented image was saved to disk and used for the CIRCOAST test.

For the in vivo images, IB4 lection was used to label the vessels, but lectin can also label activated microglia, macrophages, and the injected ASCs. However, vessel structures were differentiated from other lectin-positive cell types based on thickness, continuity of the network, and uniform edge. The images were preprocessed in an unbiased manner by loading the lectin channel in isolation into Photoshop and removing non-vessel lectin+ structures manually with the eraser tool. The images were then thresholded with a global value that was manually set for each image to capture the vascular network based on visual inspection. The binary images saved for input for the CIRCOAST test.

**Image Quantification**: colocalization was manually quantified in a blinded manner with the thresholded network channel overlaid with the channel for the cell of interest using ImageJ CellCounter Plugin (Rueden et al. 2017). Cells of interest were approximated with a circular shape based on mean area of twenty cells sampled per study group. For the in vitro experiment, live ASC cell diameter was 28.8 ± 6.0 μm and microspheres were 35.4 ± 1.2 μm. For the in vivo experiment, live ASC cell diameter was 14.7 ± 3.3 μm and dead ASC diameter was 12.1 ± 1.1 μm.

### References

Cronk SM, Kelly-Goss MR, Ray HC, Mendel TA, Hoehn KL, Bruce AC, Dey BK, Guendel AM, Tavakol DN, Herman IM, et al. 2015 Mar 13. Adipose-Derived Stem Cells From Diabetic Mice Show Impaired Vascular Stabilization in a Murine Model of Diabetic Retinopathy. Stem Cells Transl. Med. doi:10.5966/sctm.2014-0108.

Mendel TA, Clabough EBD, Kao DS, Demidova-Rice TN, Durham JT, Zotter BC, Seaman SA, Cronk SM, Rakoczy EP, Katz AJ, et al. 2013. Pericytes Derived from Adipose-Derived Stem Cells Protect against Retinal Vasculopathy. Ljubimov AV, editor. PLoS ONE 8:e65691. doi:10.1371/journal.pone.0065691.

Rueden CT, Schindelin J, Hiner MC, DeZonia BE, Walter AE, Arena ET, Eliceiri KW. 2017. ImageJ2: ImageJ for the next generation of scientific image data. BMC Bioinformatics 18:529. doi:10.1186/s12859-017-1934-z.
